# Acid ceramidase modulates the lipid profile and exacerbates sensitivity to ferroptosis in WI-38 replicative senescent cells

**DOI:** 10.1038/s41419-026-09108-y

**Published:** 2026-07-10

**Authors:** David Soriano-Castell, Marie Goujon, Nawab John Dar, Antonio Currais, Pamela Maher

**Affiliations:** Cellular Neurobiology Laboratory, The Salk Institute for Biological Studies, La Jolla, CA USA

**Keywords:** Cell death, Lipidomics

## Abstract

Cellular senescence, a complex biological process characterized by irreversible cell cycle arrest and the senescence-associated secretory phenotype, has emerged as a critical target for therapeutic development for age-related diseases. Ferroptosis, an iron-dependent regulated cell death pathway driven by the accumulation of lipid peroxidation in cell membranes, has been implicated in age-related disorders. This study investigated the relationship between cellular senescence and ferroptosis. Using human fetal lung WI-38 fibroblasts induced to senesce via replicative exhaustion, we report a novel role for acid ceramidase (ACase), which breaks down ceramides into sphingosine and free fatty acids, in regulating the sensitivity of senescent cells to RSL3-induced lipid peroxidation and ferroptosis through the modulation of polyunsaturated fatty acid composition of membrane phospholipids. Furthermore, we demonstrate a cell non-autonomous paracrine sensitization of non-senescent cells to ferroptosis by senescent cells. Together, these findings unveil ACase as a novel regulator of the ferroptosis pathway and open promising therapeutic avenues for targeting senescence-linked disorders and advancing healthy aging strategies.

## Introduction

Although emerging evidence suggests a potential link between cellular senescence and the regulated cell death pathway of ferroptosis, the molecular mechanisms underpinning this interplay remain largely unresolved [1, 2]. Ferroptosis is a non-apoptotic iron-dependent cell death pathway, also called oxytosis [3, 4], that can be induced by glutathione (GSH) depletion or the inhibition of the GSH-dependent peroxidase GPx4. These processes generate a burst of reactive oxygen species (ROS), resulting in increased peroxidation of polyunsaturated fatty acids (PUFAs) in cell membranes, which ultimately contributes to cell death [4]. Over the years, we have been using the ferroptosis pathway as a therapeutic target in a phenotypic cell-based screening platform to identify potential drug candidates for the treatment of age-related diseases [5–7]. This led to the discovery of potent anti-ferroptotic compounds, two of which (J147 and CMS121) have completed Phase 1 clinical trials for Alzheimer’s Disease [8, 9].

Despite its complex nature, the study of cellular senescence has become central in aging research [2, 10]. Notwithstanding the challenges posed by the heterogeneous senescent cell population and the absence of universal biomarkers, accumulating evidence from in vivo and in vitro studies underscores the pivotal role of senescence in various age-related disorders [11–13]. Consequently, senescent cells have become enticing targets for therapeutic interventions aimed at mitigating age-related diseases. While numerous compounds exhibit senomorphic or senolytic properties, their clinical translation requires meticulous consideration of potential off-target toxicity, especially in geropharmacology [14, 15]. Nonetheless, the promise of these interventions lies not only in their potential to prevent disease onset but also in their capacity to alleviate symptoms and promote healthy aging. Cellular senescence is characterized by irreversible cell cycle arrest triggered by prolonged stressors such as DNA damage, chemotherapy, or oxidative stress, and is accompanied by profound metabolic alterations, particularly in lipid metabolism [10, 16]. Another hallmark of senescent cells is the secretion of a complex array of inflammatory signaling molecules known as the senescence-associated secretory phenotype (SASP) [17]. The components of the SASP, which include proteins such as interleukins 1 (IL-1), 6 (IL-6), and 8 (IL-8), not only reshape local tissue microenvironments but also exert broader paracrine effects on organ function and systemic health. Moreover, this paracrine process can propagate the senescent phenotype to otherwise healthy cells, amplifying tissue dysfunction and contributing to age-related physiopathology [18, 19].

Interestingly, senescent cells display significant alterations in lipid metabolism as compared to normal cells, including accumulation of lipid droplets, increased lipid uptake, altered fatty acid (FA) composition and changes in lipid biosynthesis [20]. In addition, they present an altered membrane composition, with shifts in phospholipid profiles, increased saturation of FAs, and reduced membrane fluidity, which impair membrane signaling and contribute to their pro-inflammatory state [16, 21, 22]. Several enzymes associated with lipid metabolism have been found to be overexpressed in senescent cells, including acyl-CoA synthetase long-chain family member 4 (ACSL4) and acid ceramidase (ACase, ASAH1) [23, 24]. While ACSL4 has been implicated in ferroptosis [25, 26], the role of ACase in this type of cell death has never been explored. Extensive work in diverse systems has established that the abundance and speciation of PUFA‑containing phospholipids, shaped by enzymes such as ACSL4, are major determinants of cellular sensitivity to ferroptosis inducers [27]. Using the human fetal lung WI-38 fibroblasts model of senescence induced by replicative exhaustion, the present study shows a previously uncharacterized role for ACase in modulating ferroptosis through alterations in cellular lipid composition. We demonstrate that ACase upregulation in replicative senescence drives an increased sensitivity to ferroptosis and that inhibition of ACase expression significantly attenuates ferroptosis independently of the GPx4/GSH axis and iron regulation by decreasing the levels of PUFAs crucial for ferroptosis in membrane phospholipids, unveiling a key role for this enzyme in sensitizing replicatively senescent cells to this form of regulated cell death. Furthermore, our findings reveal a paracrine cell non-autonomous induction of ACase expression and ferroptosis sensitization in proliferative cells by the elements of the SASP, uncovering a previously unexplored relationship between senescent and proliferative cells in the context of ferroptosis with implications for aging tissue health. Together, these findings provide a further understanding of the relationship between ferroptosis and cellular senescence induced by replicative exhaustion, potentially highlighting novel therapeutic targets for the modulation or removal of senescent cells.

## Results

### Lipid peroxidation and ferroptosis sensitivity increase as cells become senescent with replicative exhaustion

**Fig. 1:**
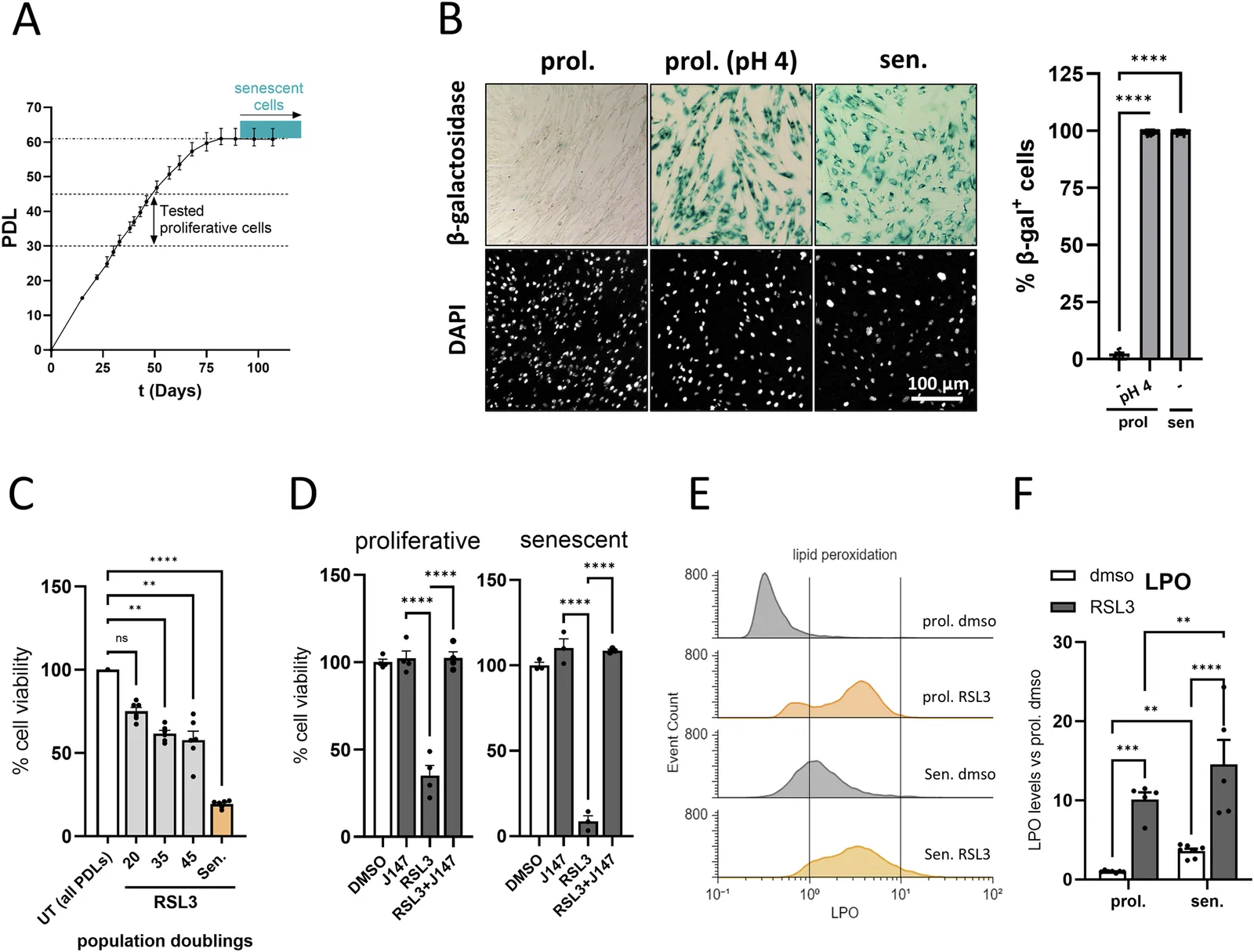
Replicative senescent cells are more sensitive to ferroptosis. **A** PDL curve of WI-38 cells indicating the stage at which proliferative and senescent cells were tested. **B** Micrographs and quantification of β-galactosidase staining of cells at the stages indicated in (**A**). Proliferative cells were also tested at pH 4 as a positive control. **C** Percentage of survival of cell populations of increasing PDL and senescent (sen.) cells after RSL3 (8 h) (250 nM) treatment. **D** Percentage of cell survival of proliferative and senescent cells after RSL3 (overnight) (250 nM) and/or J147 (1 µM) treatment (**p < 0.01, ****p < 0.0001; one-way ANOVA, multiple comparisons Tukey correction). Representative flow cytometry histograms (**E**) and quantification (**F**) showing LPO levels (C11-Bodipy 581/591) in proliferative (prol.) and senescent (sen.) cells in the presence or absence of RSL3 (4 h) (250 nM) (**p < 0.01, ***p < 0.001, ****p < 0.0001; two-way ANOVA, multiple comparisons Tukey correction). Values represent the mean ± SEM of at least three independent experiments. PDL population doubling level, LPO lipid peroxidation, prol. proliferative cells, sen. senescent cells.

Several studies have suggested a dysregulation of ferroptosis in different types of senescent cells [1, 28]. To evaluate the sensitivity of WI-38 cells to ferroptotic stress, we compared proliferative cells with senescent cells generated by replicative exhaustion. Cells became senescent after PDL60, displaying strong β-galactosidase staining, a marker of senescence (Fig. 1A, B). We treated senescent and proliferative cells of increasing population doubling levels (PDL20, PDL30 and PDL40) overnight with RSL3, an inhibitor of GPX4 and a well-known ferroptosis inducer. Survival against RSL3 treatment decreased significantly as the number of doublings increased, with the lowest level of survival observed after the cells reached senescence (Fig. 1C). In order to confirm that the cells were dying by ferroptosis, we tested the effect of J147, a compound developed in our lab based on its ability to specifically prevent ferroptosis and lipid peroxidation (LPO) [3, 29]. The effect of RSL3 was prevented by co-treating the cells with J147 (Fig. 1D), confirming that the senescent cells are undergoing ferroptosis.

Since LPO is one of the main hallmarks of ferroptosis, its levels were quantified by flow cytometry using C11-bodipy 581/591 as a probe. Consistent with the observed greater sensitivity to ferroptotic stress, senescent WI-38 cells displayed significantly higher levels of LPO as compared to replicative cells both under control conditions and when treated with RSL3 (Fig. 1E, F). Thus, there is a clear increase in LPO and ferroptosis sensitivity with the number of cellular divisions, showing maximal levels after the cells reach replicative exhaustion and senescence.

### Acid ceramidase inhibition protects senescent cells against ferroptosis

**Fig. 2:**
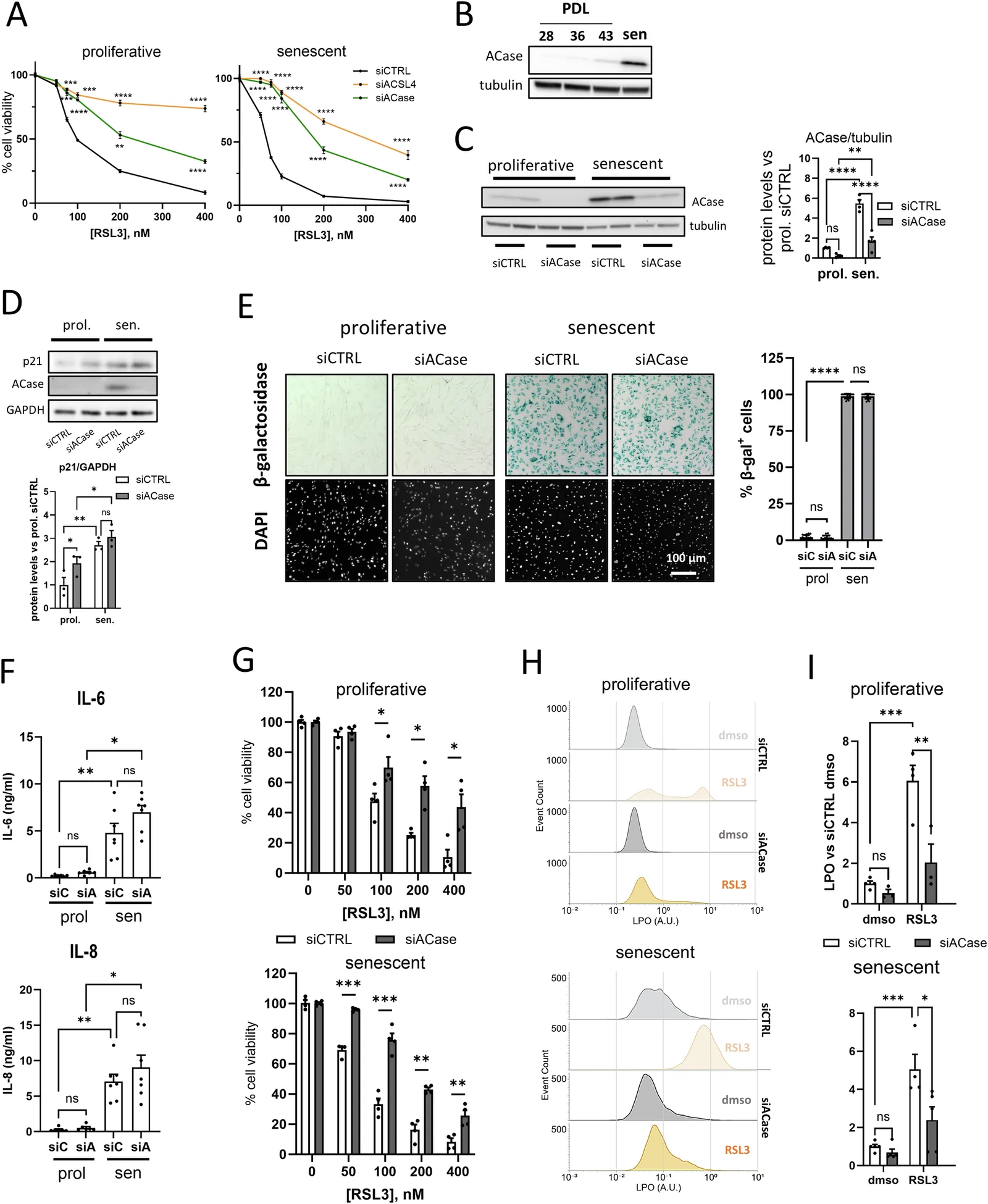
Acid ceramidase knockdown protects proliferative and senescent cells against ferroptosis. **A** Percentage of cell survival after RSL3 treatment with indicated concentrations (overnight) in the presence or absence of siACSL4 or siACase (72 h). **B** Representative blot of acid ceramidase protein expression in proliferative cells of increasing PDL and senescent cells. **C** Representative blot and quantification showing acid ceramidase knockdown after siRNA transfection (siACase). **D** Representative blot of p21 protein expression in the presence or absence of siACase. **E** Representative micrographs and quantification showing β-galactosidase activity (blue staining) before and after ACase inhibition. **F** Quantification of secreted interleukin-6 and interleukin-8 levels measured by ELISA. **G** Percentage of cell survival of proliferative and senescent cells after RSL3 treatment (overnight) in the presence or absence of siACase (72 h). **H** Representative flow cytometry histograms and quantification (**I**) showing LPO levels (C11-Bodipy 581/591) in proliferative and senescent cells in the presence or absence of RSL3 (4 h) (250 nM) and siACase (72 h) (*p < 0.05, **p < 0.01, ***p < 0.001, ****p < 0.0001; two-way ANOVA, multiple comparisons Tukey correction). Values represent the mean ± SEM of at least three independent experiments. PDL population doubling level, siC control siRNA, siA ACase siRNA, LPO lipid peroxidation.

FAs are the primary substrates of LPO [30]. Some studies have reported the overexpression of the FA-related enzymes ACSL4 and ACase in senescent cells [23, 24]. This prompted us to ask whether these enzymes are involved in the increased sensitivity to ferroptosis observed in senescent cells. In fact, the knockdown (KD) of either ACSL4 or ACase with specific siRNAs (siACSL4; siACase) strongly protected proliferative and senescent cells against RSL3 as compared to a control siRNA (siCTRL) (Figs. 2A and S1). While the role of ACSL4 in ferroptosis is well established and has been extensively described in the literature [25, 26, 31], the involvement of ACase in this type of cell death was, to our knowledge, undocumented. For this reason, we chose to concentrate our investigation on the contribution of ACase to ferroptosis sensitivity in senescent cells. ACase breaks down ceramide into sphingosine and FAs, playing a key role in lipid metabolism and affecting the abundance of specific lipidic species known to be relevant to ferroptosis (i.e., sphingomyelin, ceramide-1-phosphate or FAs) [32–34]. A strong overexpression of ACase in senescent WI-38 cells compared to replicative cells (5- to 20-fold) was confirmed by western blot (Fig. 2B). After 72 h of siACase transfection, the levels of ACase were undetectable in replicative cells and strongly, but not completely, reduced in senescent cells, consistent with previous reports showing an increased stability of ACase in senescent cells (Fig. 2C) [23]. Senescent cells transfected with siACase still displayed senescence-like phenotypes, namely, high levels of p21 expression (Fig. 2D), high levels of β-galactosidase activity (Fig. 2E), and significantly increased secretion of IL-6 and IL-8 (Fig. 2F), two of the best-known cytokines contributing to the SASP [35, 36], compared to cells transfected with siCTRL. Notably, both senescent and proliferative WI-38 cells transfected with siACase were significantly more resistant to ferroptosis induced by RSL3 as compared to cells transfected with siCTRL (Fig. 2G). The protection observed after the knockdown of ACase was additionally confirmed by treating proliferative and senescent cells with ARN14794, a specific ACase chemical inhibitor [37]. ARN14794 exerted protection against RSL3 in proliferative and senescent WI-38 cells, as well as in neuronal-derived murine HT22 cells, a well-studied cell model of ferroptosis (Fig. S2).

We then asked whether the protection by ACase KD correlated with a decrease in LPO. C11-Bodipy 581/591 was used to quantify the levels of LPO in proliferative and senescent cells transfected with siCTRL or siACase. As measured by flow cytometry, RSL3-induced LPO was significantly reduced in siACase cells compared with siCTRL cells (Fig. 2H, I) in both proliferative and senescent cells, correlating with an increased resistance to ferroptosis.

Taken together, these data indicate a clear role for ACase in sensitizing both proliferative and senescent cells to ferroptosis, with replicatively senescent cells, where the protein is strongly overexpressed, showing significantly enhanced sensitivity.

### The protection exerted by ACase inhibition is independent of both the GPX4/GSH axis and iron

**Fig. 3:**
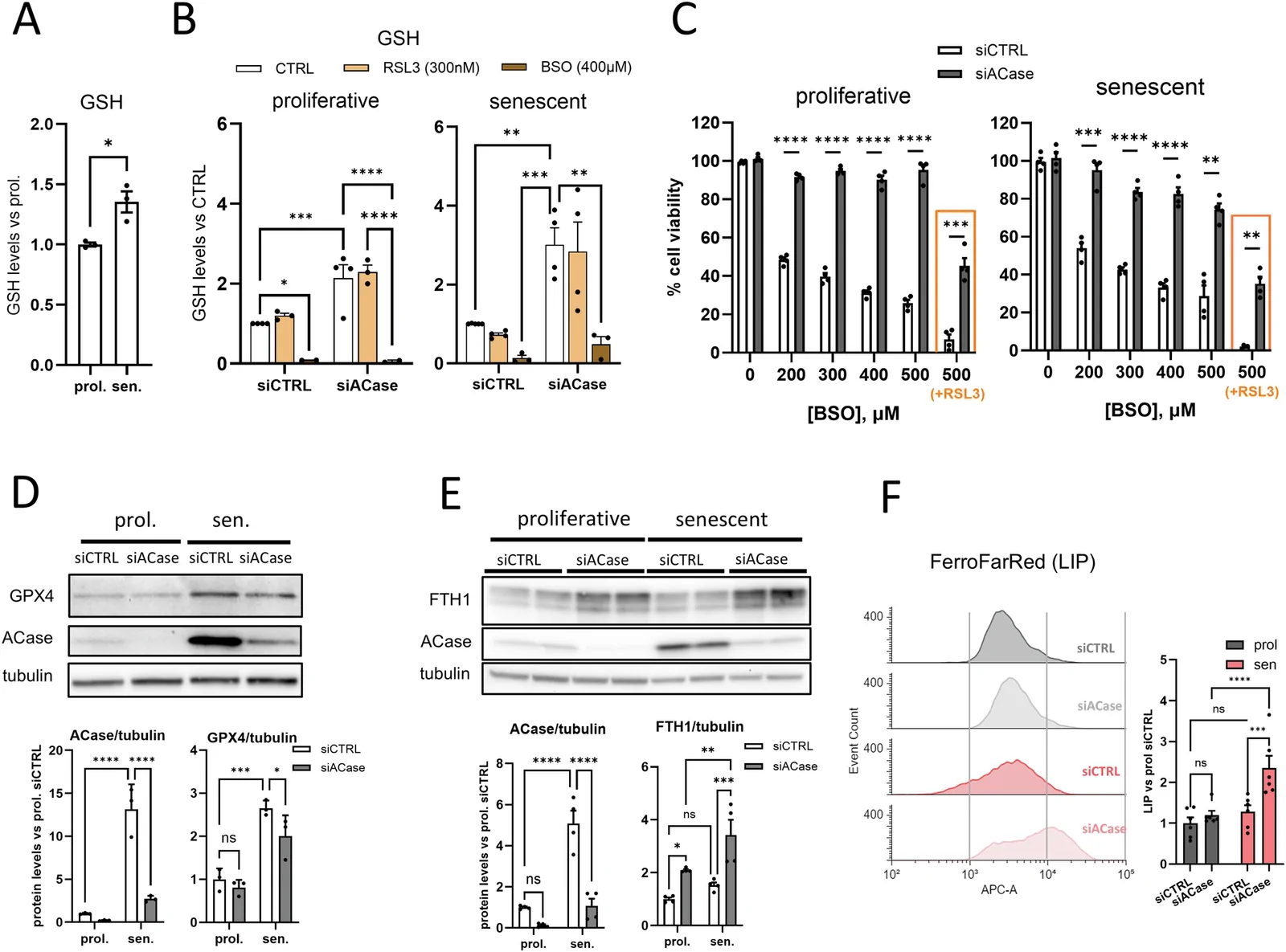
Acid ceramidase knockdown decreases lipid peroxidation independently of GSH and Fe^2+^ regulation. **A** Comparison of GSH levels between proliferative and senescent cells (*p < 0.05, paired *t*-test). **B** Quantification of GSH levels in proliferative and senescent cells after the indicated treatments. **C** Percentage of cell survival of proliferative and senescent cells after GSH depletion by BSO treatment (overnight) in the presence or absence of siACase. Co-treatment with RSL3 (overnight) is indicated in orange (200 nM). **D** Representative blot and quantification (bar graphs) of GPX4 protein expression in proliferative and senescent cells in the presence or absence of siACase. **E** Representative blot and quantification (bar graphs) of FTH1 protein expression in proliferative and senescent cells in the presence or absence of siACase. **F** Representative flow cytometry histograms and quantification (bar graph) showing labile iron pool levels (FerroFarRed) in proliferative and senescent cells in the presence or absence of siACase (*p < 0.05, **p < 0.01, ***p < 0.001, ****p < 0.0001; two-way ANOVA, multiple comparisons Tukey correction). Values represent the mean ± SEM of at least three independent experiments. prol. proliferative cells, sen. senescent cells, siCTRL control siRNA, siACase acid ceramidase siRNA, GSH glutathione, LIP labile iron pool.

To explore the anti-ferroptotic mechanism underlying the effects of ACase KD, we first determined the levels of GSH in these cells. When GSH is depleted, GPX4 cannot function effectively, and a cascade leading to increased LPO and ferroptosis is initiated [4]. Senescent cells displayed higher basal levels of GSH than proliferative cells (Fig. 3A). Notably, the silencing of ACase expression, albeit incomplete in senescent cells, induced a significant increase in total GSH levels in both proliferative and senescent cells (Fig. 3B**)**. This observation prompted us to ask whether the increase in GSH was involved in the inhibition of ferroptosis by ACase KD. To address this question, cells were treated with buthionine sulfoximine (BSO), a commonly used compound that inhibits gamma-glutamylcysteine synthetase, the rate-limiting enzyme in GSH synthesis [4]. BSO depleted GSH levels in all conditions (Fig. 3B), causing significant cell death in cells transfected with siCTRL (Fig. 3C). Strikingly, ACase KD conferred strong resistance against GSH depletion, protecting cells even after RSL3 treatment (Fig. 3C). In line with the higher levels of GSH observed in senescent cells, GPX4 protein levels were also significantly increased in these cells compared to proliferative cells (Fig. 3D). However, despite conferring a strong protection against ferroptosis, ACase KD significantly reduced GPX4 protein expression in senescent cells (Fig. 3D). These observations suggest that the protection by ACase KD is independent of the GPX4/GSH axis and that both the increase in GSH levels and the decrease in GPX4 expression could be the consequence of an attenuation of ferroptotic pressure.

Another factor that plays a critical role in ferroptosis is ferrous iron (Fe²⁺), which, through the Fenton reaction, generates hydroxyl radicals that initiate LPO and also acts as a cofactor to catalyze the enzymatic peroxidation of PUFAs by lipoxygenases (LOX) [4, 30]. The labile iron pool (LIP), which consists of loosely bound, chemically reactive Fe²⁺, is considered the primary source of iron for ferroptosis [30]. To reduce its toxicity, the ferritin protein complex buffers the excess Fe^2+^ by oxidizing and storing it as a less reactive ferric form (Fe^3+^) [38]. To explore a possible role for ferritin in ACase protection, the levels of ferritin heavy chain 1 (FTH1) were analyzed by Western blot. Notably, as seen in Fig. 3E, FTH1 levels were significantly increased after ACase KD in both proliferative and senescent cells. To test whether the observed increase in FTH1 was causing a drop in the LIP levels that could explain, at least in part, the anti-ferroptotic effect of ACase KD, the LIP levels were measured by flow cytometry using the FerroFarRed™ dye that stains Fe^2+^ but not Fe^3+^, and reflects the intracellular LIP levels [39–41]. Surprisingly, although they remained unchanged in proliferative cells, LIP levels were significantly increased in senescent cells after ACase KD, despite its anti-ferroptotic effect (Fig. 3F). This increase in LIP levels likely contributes to the observed upregulation of FTH1. The much stronger increase in Fe^2+^ levels in senescent cells as compared to proliferative cells after ACase inhibition could allow for the observable increase in LIP levels in these cells but not in proliferative cells (Fig. 3F), where the upregulation of ferritin might be sufficient to buffer a more moderate increase in Fe^2+^.

Together, these results suggest that the inhibition of ACase confers protection against ferroptosis independently of both the GPX4/GSH axis and the regulation of the intracellular Fe^2+^ levels, and that the mechanisms underlying the protection are likely upstream of these two critical elements of the ferroptosis pathway.

### ACase upregulation favors a pro-ferroptotic lipid profile in senescent cells, which is reverted after ACase inhibition

**Fig. 4:**
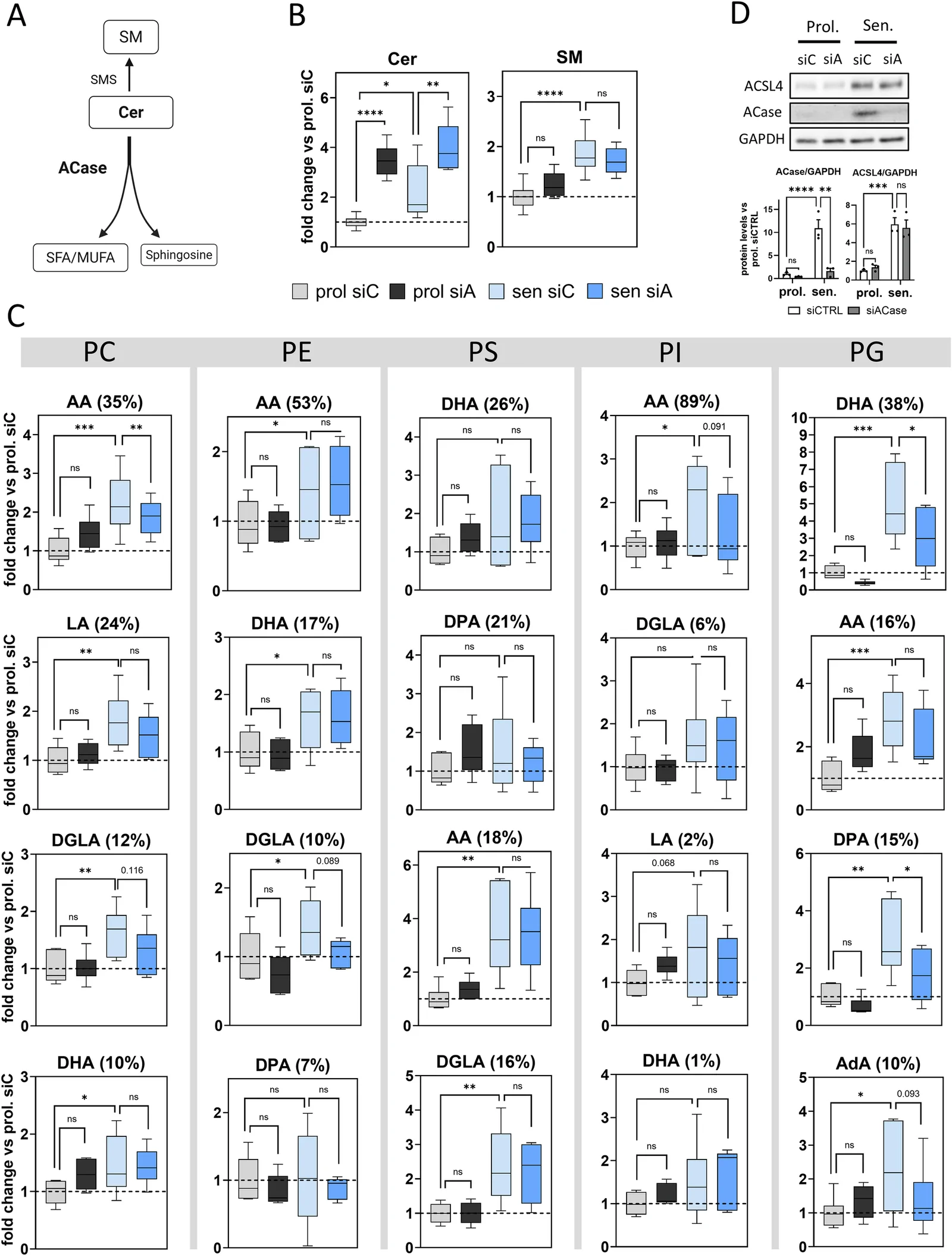
Senescent cells show a general increase in PUFA-containing phospholipids compared to proliferative cells, and this is countered by acid ceramidase inhibition. **A** Diagram representing the enzymatic relation between Cer, SM, sphingosine and FAs. **B** Relative fold change of ceramide and sphingomyelin in the indicated conditions. **C** Relative fold change of the most abundant (≥80% of total) ^sn-2^PUFA organized by PL class and ordered by abundance inside each PL class (%) from top to bottom (*p < 0.05, **p < 0.01, ***p < 0.001, ****p < 0.0001; one-way ANOVA, multiple comparisons Tukey correction). **D** Representative blot and quantification (bar graphs) of ACSL4 protein expression (**p < 0.01, ***p < 0.001, ****p < 0.0001; two-way ANOVA, multiple comparisons Tukey correction). Values represent the mean ± SEM of at least three independent experiments. Cer ceramide, SM sphingomyelin, SFA/MUFA saturated fatty acids/monounsaturated fatty acids, CerS ceramide synthase, SMS sphingomyelin synthase, prol proliferative, sen senescent, siC CTRL siRNA, siA ACase siRNA, AA arachidonic acid, DHA docosahexaenoic acid, DPA docosapentaenoic acid, DGLA dihomo-gamma-linolenic acid, LA linoleic acid, PC phosphatidylcholine, PE phosphatidylethanolamine, PS phosphatidylserine, PI phosphatidylinositol, PG phosphatidylglycerol.

The roles of the GPX4/GSH axis or iron in the execution of ferroptosis greatly depend on the dynamic regulation of the levels of membrane phospholipids (PLs), specifically those containing PUFAs (PL-PUFAs), which play a crucial role in this pathway as the substrates of LPO [42]. Comprehensive profiling of various lipidic species relevant to ferroptosis, including phosphatidylcholines (PCs), phosphatidylethanolamines (PEs), phosphatidylserines (PSs), phosphatidylglycerols (PGs), phosphatidylinositols (PI), ceramides (Cer) and sphingomyelins (SMs), showed significant differences in both the abundance and composition of these lipids between proliferative and senescent cells, both before and after ACase KD (Fig. 4).

Cer synthesis directly consumes free saturated (SFA) and monounsaturated FAs (MUFA), diverting them away from PL-PUFA production pathways [43–46]. Conversely, Cer breakdown by ACase releases FAs that can be used in lipid synthesis pathways, including those for PUFAs and PL-PUFAs [43, 47] (Fig. 4A). Interestingly, despite overexpressing ACase, senescent cells presented higher amounts of Cer species compared to proliferative cells (Figs. 4B and S3A). As expected, siACase induced an increase in Cer compared to control siRNA in both senescent and proliferative cells (Figs. 4B and S3A). Sphingomyelin (SM) species were also present at higher levels in senescent cells as compared to proliferative cells (Figs. 4B and S3A). Although most SMs are synthesized by various SM synthases using Cer as a substrate [48, 49], siACase transfection did not substantially change SM levels in senescent cells, although a slight increasing trend in proliferative cells was detected.

PUFAs in the sn-2 position (^sn-2^PUFA) of a PL are highly susceptible to LPO and are the main substrate for LPO during ferroptosis [50]. These include Omega-6 FAs like linoleic acid (LA, 18 carbons and 2 double bonds, 18:2), dihomo-gamma-linolenic acid (DGLA, 20:3), arachidonic acid (AA, 20:4) or adrenic acid (AdA, 22:4), and Omega-3 FAs like eicosapentaenoic acid (EPA, 20:5), docosapentaenoic acid (DPA, 22:5) or docosahexaenoic acid (DHA, 22:6). Although a high day-to-day variability was observed, the lipidomic analysis showed a general increasing trend in the most abundant ^sn-2^PUFA-containing PLs in senescent cells as compared to proliferative cells (Figs. 4C and S3), with significant increases in all AA-containing PLs, as well as PC-LA, PC-DGLA, PC-DHA, PE-DHA, PE-DGLA, PS-DGLA, PG-DHA and PG-AdA. Importantly, ACase-KD senescent cells displayed a general decrease across the most abundant PL-PUFA species compared to control siRNA senescent cells (Figs. 4C and S3). This effect was statistically significant for PC-AA, PG-DHA and PG-DPA and showed a strong trend for PC-DGLA (*p* = 0.116), PE-DGLA (*p* = 0.089), PI-AA (*p* = 0.091), and PG-AdA (*p* = 0.093) (Figs. 4C and S3). Changes in PL-PUFAs were less consistent in proliferative cells after siACase transfection, possibly due to the lower basal levels combined with a high day-to-day variability. A significant increase in PL containing SFA/MUFA was also observed in senescent cells as compared to proliferative cells, especially PC-, PS- and PG-SFA/MUFA (Fig. S3G). Additionally, albeit statistically non-significant, siACase senescent cells displayed a decreasing trend in the levels of PL-SFA/MUFA compared to siCTRL cells.

ACSL4 is a well-known driver of PUFA esterification and incorporation into PL-PUFAs in cell membranes, and it plays a critical role in the execution of ferroptosis [25]. Therefore, we asked whether the decrease in PL-PUFAs observed in ACase KD senescent cells could be the consequence of a possible indirect inhibition of ACSL4 expression. As shown in Fig. 4D, siACase transfection did not alter ACSL4 protein expression in either proliferative or senescent cells, suggesting that the observed reduction in PL-PUFA levels following ACase KD occurs independently of ACSL4.

Taken together, these data demonstrate that knockdown of ACase increases the Cer pool, and thereby the number of FAs incorporated into Cer, which could decrease the overall number of FAs available for the synthesis of PUFAs and their incorporation into membrane PLs. The decrease in the most abundant PL-PUFAs observed after ACase KD supports the hypothesis that ACase upregulation after reaching replicative senescence favors a pro-ferroptotic lipid profile compared to proliferative cells and could explain the increased sensitivity to ferroptosis of senescent cells and the protection by ACase inhibition by decreasing the levels of LPO substrates in cell membranes.

### Cell non-autonomous cytokine signaling from replicatively senescent cells increases ACase expression and ferroptosis sensitivity in proliferative cells

**Fig. 5:**
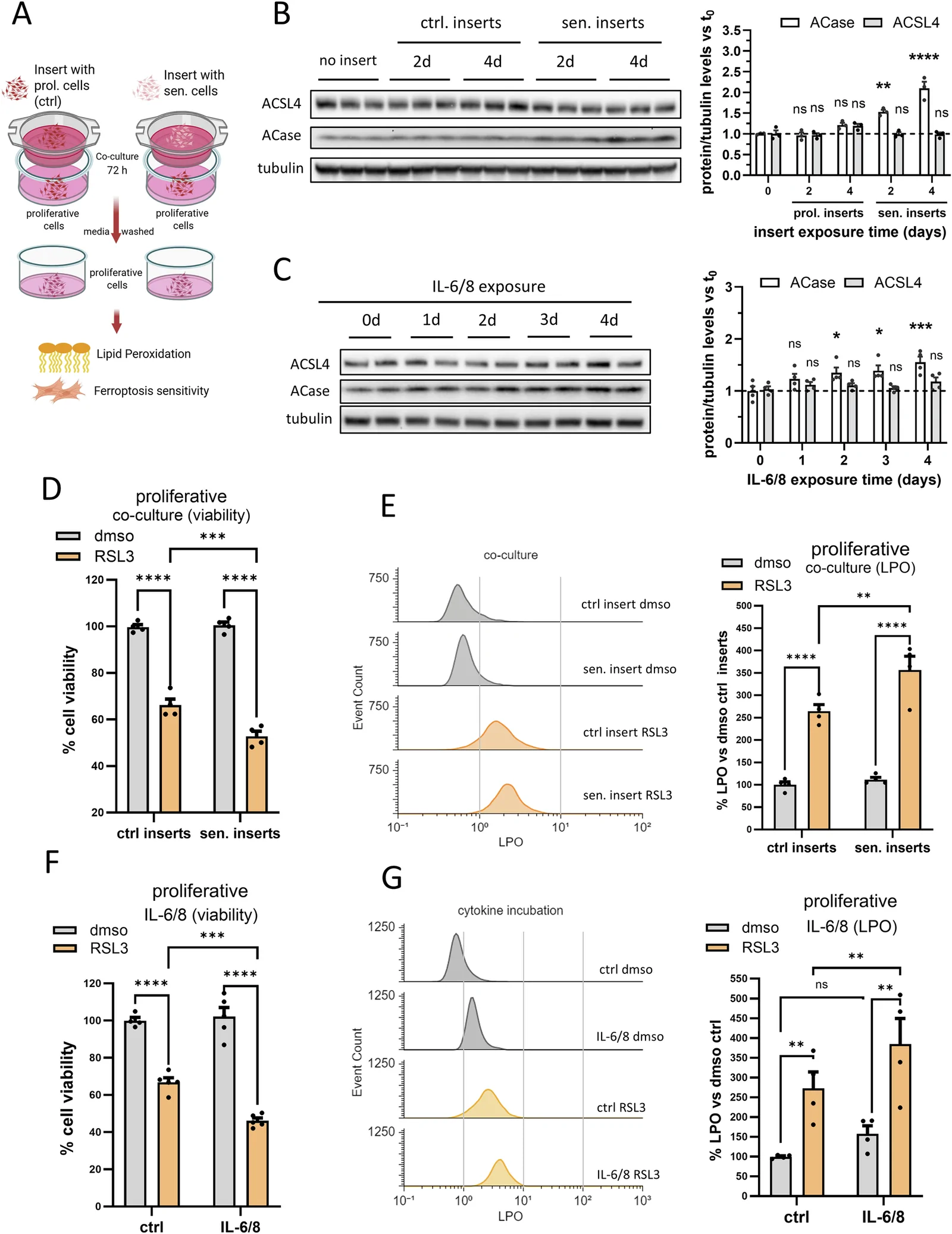
Paracrine signaling from senescent cells increases ACase expression and ferroptosis sensitivity in proliferative cells. **A** Diagram illustrating the co-culture protocol. **B** Representative blot and quantification (bar graphs) of ACase and ACSL4 protein expression in proliferative cells (PDL 35–45) co-cultured with inserts as in (**A**) for the indicated times (**p < 0.01, ****p < 0.0001; two-way ANOVA, multiple comparisons Tukey correction). **C** Representative blot and quantification (bar graphs) of ACase and ACSL4 protein expression of proliferative cells incubated with IL-6/8 (50/25 ng/ml) for the indicated times (*p < 0.05, ***p < 0.001; two-way ANOVA, multiple comparisons Tukey correction). **D** Percentage of cell survival of proliferative cells after RSL3 treatment (overnight) (250 nM) in the presence or absence of senescent cells. **E** Representative flow cytometry histograms and quantification (bar graphs) showing LPO levels (C11-Bodipy 581/591) in proliferative cells after RSL3 treatment (4 h) (250 nM) in the presence or absence of senescent cells. **F** Percentage of cell survival of proliferative cells after RSL3 treatment (overnight) (250 nM) in the presence or absence of IL-6/8 (72 h). **G** Representative flow cytometry histograms and quantification (bar graphs) showing LPO levels (C11-Bodipy 581/591) in proliferative cells after RSL3 treatment (4 h) (250 nM) in the presence or absence of IL-6/8 (**p < 0.01, ***p < 0.001, ****p < 0.0001; two-way ANOVA, multiple comparisons Tukey correction). Values represent the mean ± SEM of at least three independent experiments. prol. proliferative cells, sen. senescent cells, LPO lipid peroxidation.

It is well known that senescent cells can affect healthy neighboring cells within the reach of the paracrine effect of the SASP, inducing certain senescent-like phenotypes in proliferative cells [51]. To explore whether the changes in the regulation of ACase and ferroptosis sensitivity that we found in senescent cells could indeed spread to non-senescent cells, proliferative cells were incubated for 2 and 4 days with inserts containing proliferative or senescent cells, allowing for media sharing without direct cell contact (Fig. 5A). After the incubation, the inserts were removed, and the proliferative cells were analyzed. Interestingly, while the expression levels of ACSL4 were not affected, the co-culture with senescent cells induced a significant time-dependent increase in ACase expression as compared to proliferative cells incubated with no inserts or with inserts containing other proliferative cells (Fig. 5B). Importantly, a similar effect was observed after the incubation of proliferative cells with a combination of recombinant human interleukin-6 and interleukin-8 (IL-6/8), two of the most representative SASP cytokines that are often used synergistically to mimic the paracrine effect of the SASP and other inflammatory signals [52–54] (Fig. 5C). We next asked whether the co-culture with senescent cells or the incubation with cytokines from the SASP could increase the sensitivity of proliferative cells to RSL3-induced LPO and ferroptotic cell death. Figure 5D shows a small but significant decrease in the viability of proliferative cells co-cultured with senescent cells after RSL3 treatment as compared with cells co-cultured with other proliferative cells. As expected, this decrease in cell viability correlated with significantly elevated levels of LPO, as measured by flow cytometry (Fig. 5E). This paracrine effect of senescent cells on the sensitivity to RSL3 and LPO levels of proliferative cells was supported by incubating the proliferative cells with the SASP components IL-6/8 (Fig. 5F, G).

Overall, these data suggest that the paracrine effect of cytokines such as IL-6/8 secreted by replicatively senescent cells induces senescent-like features in proliferative cells, including increased ACase protein levels and exacerbation of LPO production, that can sensitize them to ferroptosis.

## Discussion

**Fig. 6:**
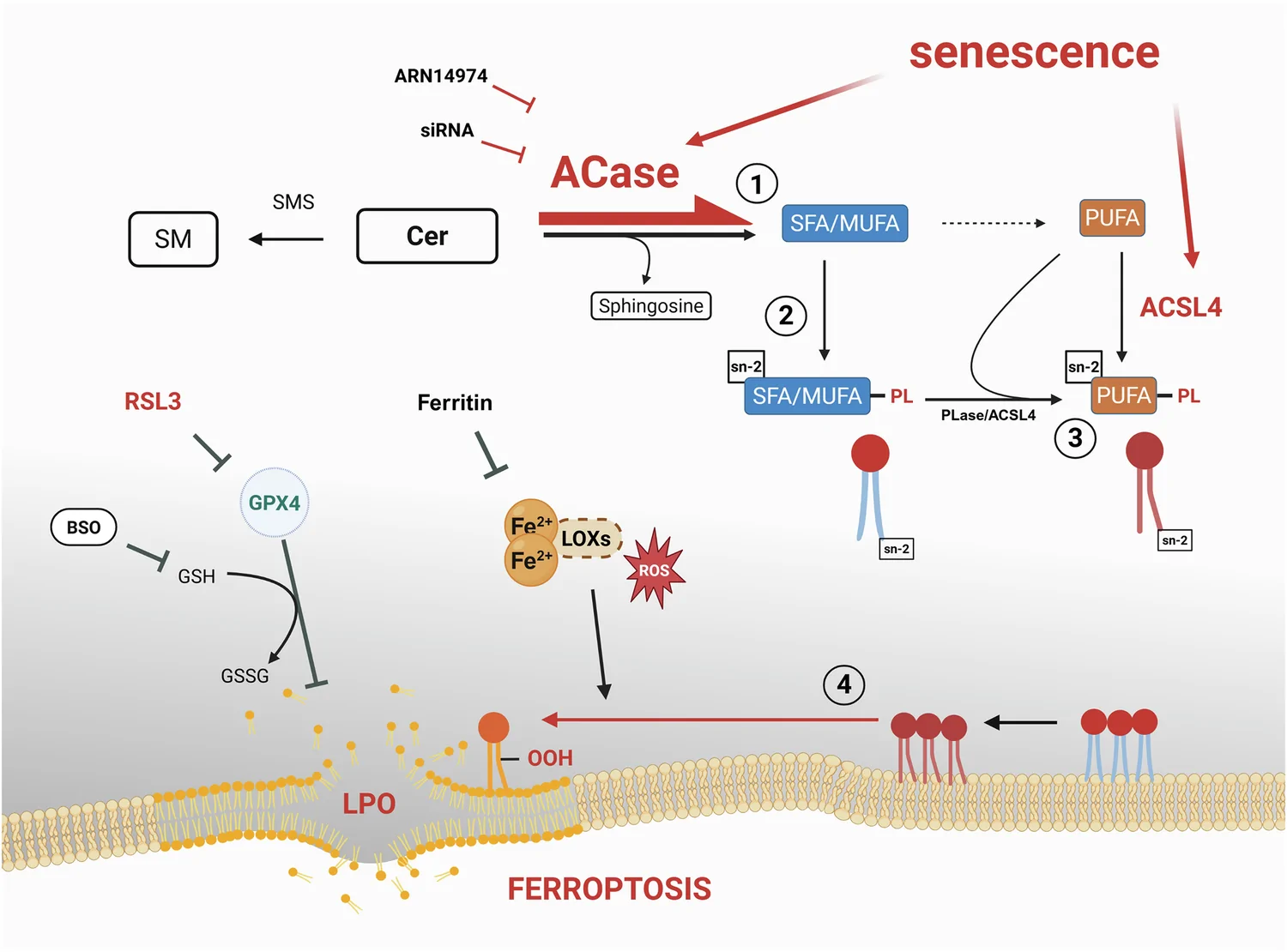
Working model illustrating the possible interplay between ACase and ferroptosis in senescent cells. The upregulation of ACase in senescent cells would increase the pool of SFA/MUFA from the Cer breakdown (1). Through the Kennedy pathway (2) and/or the Lands cycle (3), increased ^sn-2^SFA/PUFA-PL would lead to an accumulation of ^sn-2^PUFA-PL, priming the cell membranes for LPO and ferroptosis (4). SM sphingomyelin, Cer ceramide, SFA saturated fatty acid, MUFA monounsaturated fatty acid, PUFA polyunsaturated fatty acid, PL phospholipid, ROS reactive oxygen species, LPO lipid peroxidation, GSH reduced glutathione, GSSG oxidized glutathione.

Although the high-PDL WI-38 cultures used in this study represent an advanced stage of senescence driven by replicative exhaustion that may occur only in a small subset of cells in vivo, this canonical, well-defined model is able to consistently recapitulate key SASP and metabolic reprogramming signatures shared with other senescence models both in vitro and in vivo and thus provides a homogeneous, mechanistically well-defined context in which to interrogate ferroptosis-related metabolic alterations in senescent cells [55–57]. In addition, the embryonic origin and low in vivo division history of this line, together with its widespread use as a stable model for replicative senescence, reduce the confounding effects of prior proliferative and stress exposures and the heterogeneity typically observed in adult primary fibroblasts [58, 59]. Mixed reports on ferroptosis in senescent cells can be found in the literature. Several studies have reported higher resistance of some senescent cells to ferroptotic cell death due to disrupted ferritin and iron regulation [28, 60, 61]. On the other hand, recent reports show a pro-ferroptotic signaling activated upon senescence in vascular smooth muscle cells and an increased sensitivity to ferroptosis of senescent kidney tubular cells [62, 63]. Consistent with the latter reports, our results clearly indicate that replicative senescent human embryonic WI-38 lung fibroblasts have elevated LPO and higher sensitivity to ferroptosis upon RSL3 treatment, as compared to proliferative cells. Senescent cells can display elevated PUFA content and dysregulated lipid homeostasis as a consequence of their metabolic reorganization, which may drive their enhanced susceptibility to ferroptosis [51, 64]. Here we show that ACase upregulation in replicative senescent cells plays a significant role in creating a pro-ferroptotic lipid profile that can help to explain the observed higher sensitivity to ferroptosis (Fig. 6). While ferroptosis sensitivity in senescent cells may vary depending on the senescence trigger or cell line, our results indicate that ACase overexpression, along with the associated lipid metabolism shifts observed here, drives increased sensitivity to ferroptotic insults relative to replicative cells. We therefore predict that senescence would be pro-ferroptotic in other contexts where similar ACase upregulation and lipid profile changes occur.

Our results reveal that the protection exerted by ACase KD is independent of GSH. Similarly, the modulation of LIP levels does not play a role in this protection, since labile Fe^2+^ is significantly elevated after ACase inhibition, which is typically a pro-ferroptotic feature. Interestingly, the reports showing a higher resistance of senescent cells to ferroptosis also report a strong accumulation of iron [28, 60, 61]. GSH participates as a key part of the enzymatic defense against LPO via the action of GSH-peroxidases such as GPX4, which use GSH to reduce lipid hydroperoxides [30]. In this context, GSH is depleted as it is consumed, hence decreases in LPO are often a consequence of higher GSH availability. However, although we observed a steep increase in GSH levels after the inhibition of ACase, our data show that siACase protects equally well against ferroptosis in GSH-depleted cells. This is consistent with the idea that ACase KD induces an anti-ferroptotic state where there is less demand for GSH-dependent antioxidant activity. Such a state could be driven by a decrease in the PUFA content of the cell, which would result in less substrate for LPO, decreasing the oxidative burden on GSH and GSH-dependent enzymes [65, 66]. Therefore, the elevated GPX4 levels observed in senescent cells likely reflect a compensatory response to the chronically increased LPO substrate burden, as evidenced by the significant decrease in GPX4 expression following ACase knockdown and the associated reduction in LPO. Whether basal GPX4 and ACase expression levels could predict sensitivity to different ferroptotic triggers, and to what extent this is cell line-specific, remains an interesting open question for future investigation.

When synthesized de novo, membrane phospholipids are initially formed with SFAs or MUFAs at the sn-1 and sn-2 positions, often using FAs derived mainly from the pool of free SFAs and MUFAs in the cell in a process known as the Kennedy pathway [67–69] (Fig. 6). These free FAs are activated to acyl-CoAs by SFA/MUFA-specific acyl-CoA synthetases (e.g., ACSL enzymes) before being added to PLs [70, 71]. Through the action of phospholipases like PLA2 that primarily remove the ^sn-2^FAs and the activation of free PUFAs by ACSL4, the ^sn-2^SFA- and ^sn-2^MUFA-PLs are remodeled into ^sn-2^PUFA-PLs, a pathway known as the Lands cycle [31, 72]. The cellular pool of free SFA/MUFAs can originate from de novo lipogenesis, exogenous intake or lipolysis of stored lipids such as ceramides [72]. The strong overexpression of ACase observed in senescent cells, along with an increase in total ceramides compared to proliferative cells (Fig. 4B), could indeed amplify the release of free SFA/MUFAs by the lipolysis pathway. Higher levels of SFA/MUFA-PLs can confer resistance against ferroptosis in some contexts by competing with PUFAs for membrane incorporation, making membranes more rigid and less prone to oxidation [27, 73]. However, their excess has also been reported to promote PUFA-PL accumulation through the Lands cycle exchange, increasing vulnerability to peroxidation and ferroptosis [27, 74]. Consistent with this scenario, we observed increased levels of both SFA/MUFA-PLs and PUFA-PLs in senescent cells along with higher LPO levels (Fig. 6).

PC and PG displayed the most significant changes between proliferative and senescent cells, as well as after ACase inhibition. PC is the most abundant PL in eukaryotic membranes, including the mitochondria and the outer leaflet of the plasma membrane, and ^sn-2^PUFA-PCs are major contributors to ferroptosis [72]. Conversely, PG is a less abundant PL, and its contribution to ferroptosis is largely unknown. However, direct strong oxidation of ^sn-2^PUFA-PGs during ferroptosis has been reported [75]. PGs are highly enriched in mitochondria, especially the inner membrane, where they are rapidly transformed into cardiolipin (CL) and support protein translocation or respiratory chain activity [76]. PUFA-CL peroxidation has been proposed as a marker of mitochondrial LPO [77]. Given the critical role of mitochondria during ferroptosis [78, 79], the role of ^sn-2^PUFA-PGs, and possibly CL, could be of special relevance in the context of senescence and warrants further investigation.

ACSL4 plays a critical role in the regulation of PL-PUFA composition [25, 80, 81]. Numerous studies have demonstrated that ACSL4 is critical for the execution of ferroptosis, and it is upregulated during senescence [24, 25]. High ACSL4 expression increases the PL-PUFA content, and thus the readily oxidizable lipid pool and ferroptosis sensitivity. In fact, ACSL4-KD protects senescent cells against RSL3 in our model. Despite the observed decrease in PL-PUFA content and LPO after ACase inhibition, our results showed that ACSL4 protein levels remain high in senescent cells following ACase inhibition, suggesting that the downregulation of the SFA/MUFA-PLs supply could play a role in the decrease of PUFA-PLs independently of ACSL4. In addition, the increase in ceramide content observed after ACase KD could also limit the pool of available free SFAs and MUFAs for other anabolic lipid processes, including elongation/desaturation and PUFA re-acylation by ACSL4 [72]. Nonetheless, while our findings show a strong association between ACase inhibition and decreased membrane PL-PUFA levels, future studies should further explore the underlying biochemical pathways driving this effect.

The SASP can induce paracrine effects, influencing nearby cells, thereby altering the local tissue environment [17, 36, 82]. One prominent paracrine effect is the induction of senescence in neighboring healthy, non-senescent cells, a process known as paracrine or bystander senescence [83]. This occurs through SASP factors that trigger DNA damage responses or activate pathways that lead to senescence, thereby potentially amplifying tissue dysfunction over time. Here, we found that WI-38 replicatively senescent cells can induce a significant increase in ACase expression in proliferative cells through the SASP, with IL-6 and IL-8 being sufficient to exert this response. Importantly, we observed a significant increase in the levels of LPO and the sensitivity to ferroptotic cell death of non-senescent cells after IL-6/8 incubation or co-culture with senescent cells. Although we did not explore the long-term effects of the SASP on proliferative cells in the present study, which could eventually induce secondary senescence, our findings further highlight the potential damage through propagation that can arise from the accumulation of replicatively exhausted cells during disease or aging, triggering not only senescence of bystander cells but also increased sensitivity to ferroptotic death.

Growing evidence indicates that a gradual increase in ferroptotic-related stress, even in the absence of cell death, can be a consequence of the aging process itself [84]. For this reason, eliminating senescent cells may in turn contribute to a general reduction in the ferroptotic-related stress of aging tissues. Indeed, several studies have explored the increased susceptibility of senescent cells to ferroptosis as a strategy for their clearance [85]. However, our data indicates a further complexity in the interplay between cell survival, senescence and cancer. For example, ACase has been shown to promote the survival of senescent cells by controlling ceramide levels and increasing sphingosine and sphingosine-1-phosphate (S1P) levels, both of which are associated with cell survival and reduced apoptosis [23]. This primary metabolic shift could be causing susceptibility to ferroptosis as an unintended secondary effect. Conversely, ACase activity has been found to contribute to tumorigenesis in melanoma cells and to confer resistance to radiotherapy in prostate cancer cells [86, 87], and ACase is overexpressed in various malignancies [88–90]. As a matter of fact, cancer cells often display a higher susceptibility to ferroptosis, due to their elevated iron content, high oxidative stress and altered lipid metabolism, and numerous studies have explored the induction of ferroptosis as an approach to treating cancer for this reason [91, 92]. In addition, it is worth noting that, while replicative senescence appears to sensitize WI-38 cells to ferroptosis, several reports have shown an increased resistance of these and other senescent cells to apoptotic cell death after replicative exhaustion [93, 94]. Therefore, further studies exploring in depth the modulation of ACase and the different types of cell death in the context of aging may be highly valuable.

Altogether, the present study provides not only further evidence for an increased sensitivity of senescent cells to ferroptosis but also a deeper understanding of the mechanisms underlying this change. In addition to expanding our knowledge about how changes in lipid metabolism associated with replicative senescence can influence the sensitivity to cell death, these findings open new avenues for the development of effective senolytic drugs that attenuate or counteract the deleterious effects of senescent cells during aging.

## Materials and methods

### Materials

ELISA kits for IL-6 (#88-7066-77) and IL-8 (#88-8086-77) were purchased from Life Technologies. Human IL-6 (#20006) and IL-8 (#20008) recombinant proteins were purchased from PeproTech. Ferroptosis inducer RSL3 (#HY-100218A) was purchased from MedChemExpress. Buthionine-sulfoximine (BSO) was purchased from Sigma (#B2515); ARN14794 was purchased from Cayman Chemical (#17119).

### Cell cultures and replicative senescence

Human WI-38 embryonic lung fibroblasts (Coriell Institute) and HT22 mouse hippocampal nerve cells were cultured in high-glucose Dulbecco’s modified Eagle’s medium (#11995065, Invitrogen) supplemented with 10% fetal bovine serum (FCS) (#SH30073, Hyclone) and incubated at 37 °C in 10% CO_2_. WI-38 cells with a population doubling level (PDL) of 20 were thawed and grown under standard conditions. Cells were counted and passaged when they reached 70–80% confluency. Cells stopped dividing and became senescent at PDL 55–60. Cells ranging between PDL30 and PDL40 were used as control proliferative cells. After the cells stopped dividing, senescence-associated β-galactosidase staining (#9860, Cell Signaling), p21 protein levels and analyses of culture supernatants to detect the factors of the SASP IL-6 and IL-8 were performed to characterize replicative senescent cells.

### Ferroptosis in vitro assay

5 × 10^3^ HT22 or WI-38 cells were plated per well in 96-well plates and incubated overnight. The medium was then exchanged with fresh medium, and RSL3 (200 nM, unless otherwise indicated) was added alone or in combination with the indicated compounds at the indicated concentrations. 24 h later, the cellular viability was measured by the 3-(4, 5-dimethylthiazolyl-2)- 2,5-diphenyltetrazolium bromide (MTT) assay, as previously described [95]. The MTT method was validated by correlation with propidium iodide and DAPI staining assessed by flow cytometry and fluorescence microscopy (Fig. S4).

### Measurement of lipid peroxidation and labile Fe^2+^ levels by flow cytometry

7.5 × 10^4^ WI-38 cells per well were seeded in 24-well plates. After 24 h, the indicated compounds were added, and after 3–6 h, the media was aspirated, and 250 μl per well of BODIPY 581/591 C11 (#D3861, Invitrogen) (1 μM) or FerroFarRed (#SCT037, Goryo Chemical) (5 μM) were added in the presence of the different compounds, to detect LPO or labile Fe^2+^, respectively. The cells were incubated for 30 min, washed and trypsinized. The fluorescence was measured at 4 °C using a FACSymphony A3 flow cytometer and the BD FACSDiva software (BD Biosciences, USA). The FerroFarRed signal was measured with the APC Diva parameter. LPO was calculated ratiometrically by dividing the green signal (BB515 Diva parameter) by the red signal (PE Diva parameter); hence, the levels of LPO were directly proportional to the green fluorescence and inversely proportional to the red fluorescence. The data were normalized to the emission of control cells treated with DMSO.

### Measurement of glutathione levels

For measurement of total glutathione (tGSH), 3 × 10^5^ WI-38 cells were plated in 60 mm dishes. After 24 h, the medium was exchanged with fresh medium, and corresponding treatments were added for 4 h. After this, the cells were scraped into ice-cold PBS, and 10% sulfosalicylic acid was added at a final concentration of 3.3%. tGSH was determined by the recycling assay based on the reduction of 5,5-dithiobis (2-nitrobenzoic acid) with glutathione reductase and NADPH [96], and normalized to protein recovered from the acid-precipitated pellet by treatment with 0.2 N NaOH at 37 °C overnight and measured by the bicinchoninic acid assay (#23225, Pierce).

### Western blotting

Cells were washed and scraped into cold PBS. The supernatant was discarded, and cells were lysed in RIPA lysis buffer (Santa Cruz Biotechnology) containing protease/phosphatase inhibitors (Bio-Rad) and incubated on ice for 10 min. Lysates were separated by SDS-polyacrylamide gel electrophoresis (SDS-PAGE) and transferred onto PVDF membranes via semi-dry transfer (Trans-Blot® Turbo, Bio-Rad) and immunoblotted with the respective antibodies (ASAH1 #sc-136275, Santa Cruz; ACSL4 # sc-271800, Santa Cruz; FTH1 #4393, Cell Signaling; GPx4 #sc-166570, Santa Cruz; p21, #37543 Cell Signaling), followed by incubations with the appropriate secondary antibodies conjugated with horseradish peroxidase (Bio-Rad). Levels of the protein of interest were normalized to housekeeping proteins (tubulin #2148, Cell Signaling; GAPDH #97166, Cell Signaling).

### siRNA transfection

Cells were seeded in 100 mm dishes at a density of 2 × 10^4^ cells. 24 h post-seeding, cells were transfected with siRNA against acid ceramidase (ACase) (ASAH1, #sc-105032, Santa Cruz) or a negative control siRNA (#1027280, Qiagen), using lipofectamine RNAiMAX (#13778075, ThermoFisher) as the transfection reagent and Opti-MEM (#31985070, Gibco) as the transfection medium. Medium was replaced 24 h post-transfection, and the cells were incubated in fresh media for 72 h. Transfection efficiency was determined via Western blot.

### Lipidomics

After 72 h of control or ACase siRNA transfection, cells were trypsinized, washed and centrifuged, and the pellet was flash-frozen with liquid nitrogen and stored at -80˚C. Lipidomic analysis was performed by the LIPID MAPS Lipidomics Core at the University of California, San Diego, following established protocols [97]. The analysis focused on phospholipids, ceramides, sphingomyelins, and sphingoid bases. Lipids were extracted from samples using a modified Folch method with chloroform/methanol (2:1, v/v). The extracted lipids were then analyzed using a combination of shotgun and LC-based lipidomics approaches. Data processing and quantification were performed using specialized software, with multiple standards, including internal standards and endogenous abundance species, employed for accurate quantitation. The analysis provided comprehensive profiling and quantification of the lipid species present in the samples. Statistical and pathway analyses were performed using Python and GraphPad software.

### Cytokines and co-culture experiments

1.5 × 10^4^ proliferative cells (PDL 40–45) per well were seeded in 24-well plates. After attachment, the cells were incubated for 96 h with a combination of IL-6/IL-8 (2:1 ratio, 50/25 ng/ml), based on previous reports of synergistic IL-6 and IL-8 paracrine effects [52–54, 98–100]. For the co-culture experiments, inserts (#662641, Greiner) seeded with 4.5 × 10^4^ senescent cells or 1.5 × 10^4^ proliferative cells (control) were placed in the wells instead of IL-6/8. After the treatments, the media with cytokines or the inserts were removed, and the remaining cells in the well were washed before being tested for protein analysis, cell survival or LPO quantification.

### Quantification and statistical analysis

Statistical analysis was performed using GraphPad Prism 8, and a *p* value of <0.05 was considered as significant. The data representation and statistical tests applied are described in the figure legends and supplementary material Fig. S5 and Table S1. Homogeneity of variance across groups was confirmed using Levene’s test prior to ANOVA analyses. For two-way ANOVA, residual plots were inspected to verify that equal variance assumptions were met. Post-hoc power analysis was performed using GPower 3.1, confirming ≥80% power for all primary comparisons at *α* = 0.05.

## Supplementary information

Supplementary Figures

Editable supplementary Table S1

Uncut WBs

## Data Availability

All data generated or analyzed during this study are included in this article and its supplementary information files or are available from the corresponding authors upon request.
