# Supplementary material for: Acid ceramidase modulates the lipid profile and exacerbates sensitivity to ferroptosis in WI-38 replicative senescent cells
Source: Cell Death Dis. 2026 Jul 10;17(1):860. doi: 10.1038/s41419-026-09108-y (PMC13638989; doi:10.1038/s41419-026-09108-y)
Supplement: Supplementary file 1 [file 41419_2026_9108_MOESM1_ESM.pdf]

Supplementary Fig. S1

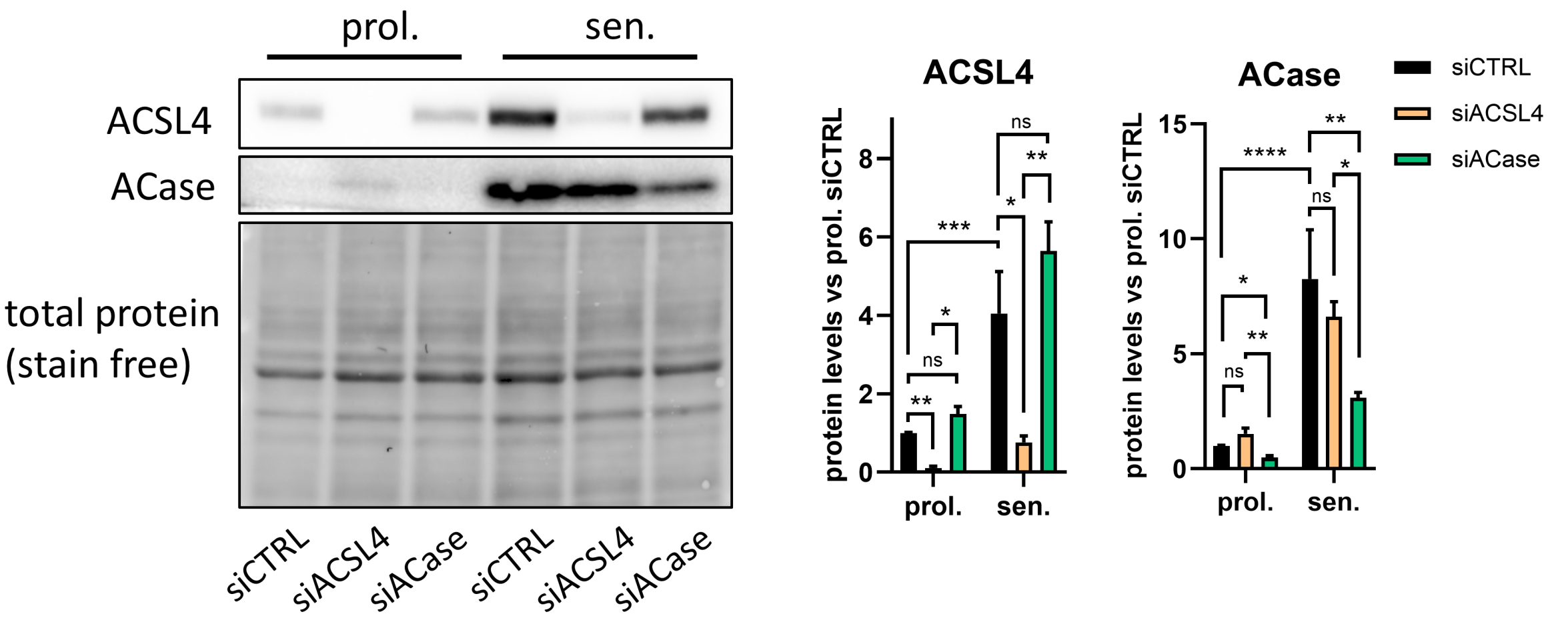

Figure S1. Silencing of ACSL4 and ACase by siRNA transfection in proliferative and senescent WI-38 cells. Proliferative and senescent cells were transfected with specific siRNAs targeting ACSL4 and ACase for 72 hours to test the role of these proteins in ferroptosis sensitivity (see Fig. 2A). ($^{*}p < 0.05$, $^{**}p < 0.01$, $^{***}p < 0.001$, $^{****}p < 0.0001$. Two-way ANOVA). Values represent the mean ± SEM of 3 independent experiments. prol, proliferative; sen, senescent.

Supplementary Fig. S2

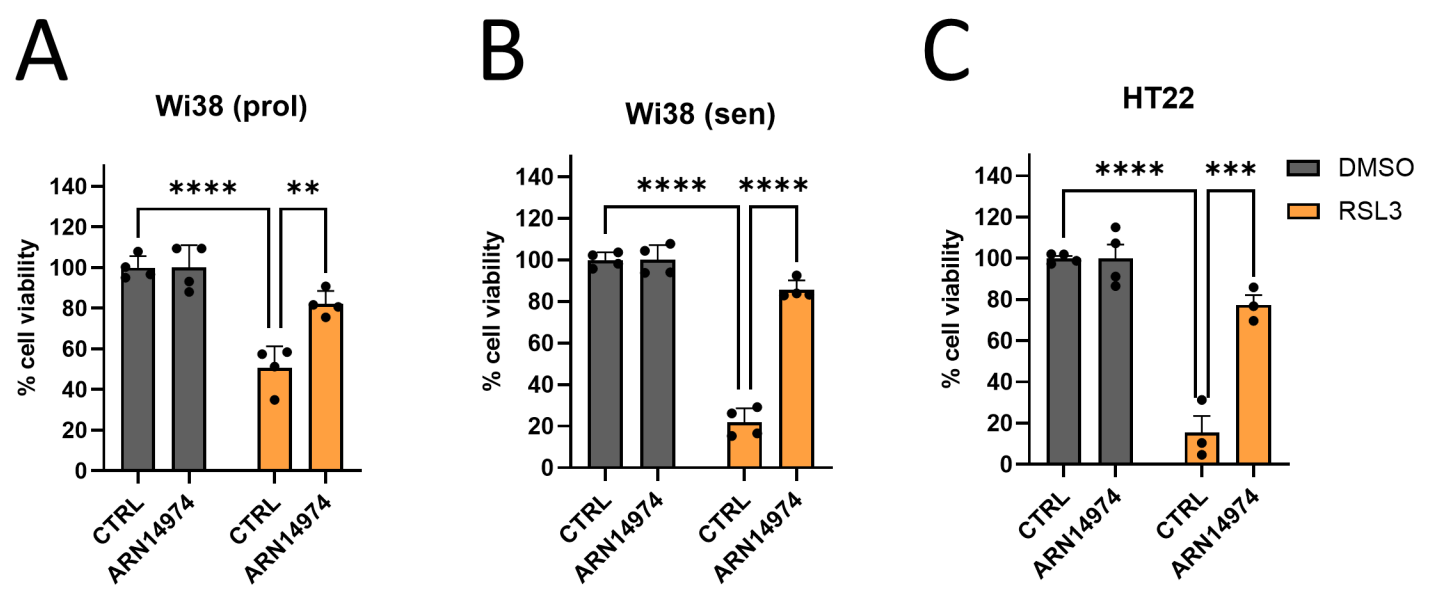

Figure S2. ACase specific chemical inhibitor ARN14794 protects both human Wi38 and murine HT22 cells. Percentage of cell survival of Wi38 proliferative (PDL 35-45) (A) or senescent (B) cells and HT22 cells (C) against ferroptosis inducer RSL3 (100 nM) overnight in the presence or absence of the ACase inhibitor ARN14794 (25 µM). ($^{**}p < 0.01$, $^{***}p < 0.001$, $^{****}p < 0.0001$. Two-way ANOVA). Values represent the mean ± SEM of at least 3 independent experiments. prol, proliferative; sen, senescent.

Supplementary Fig. S3

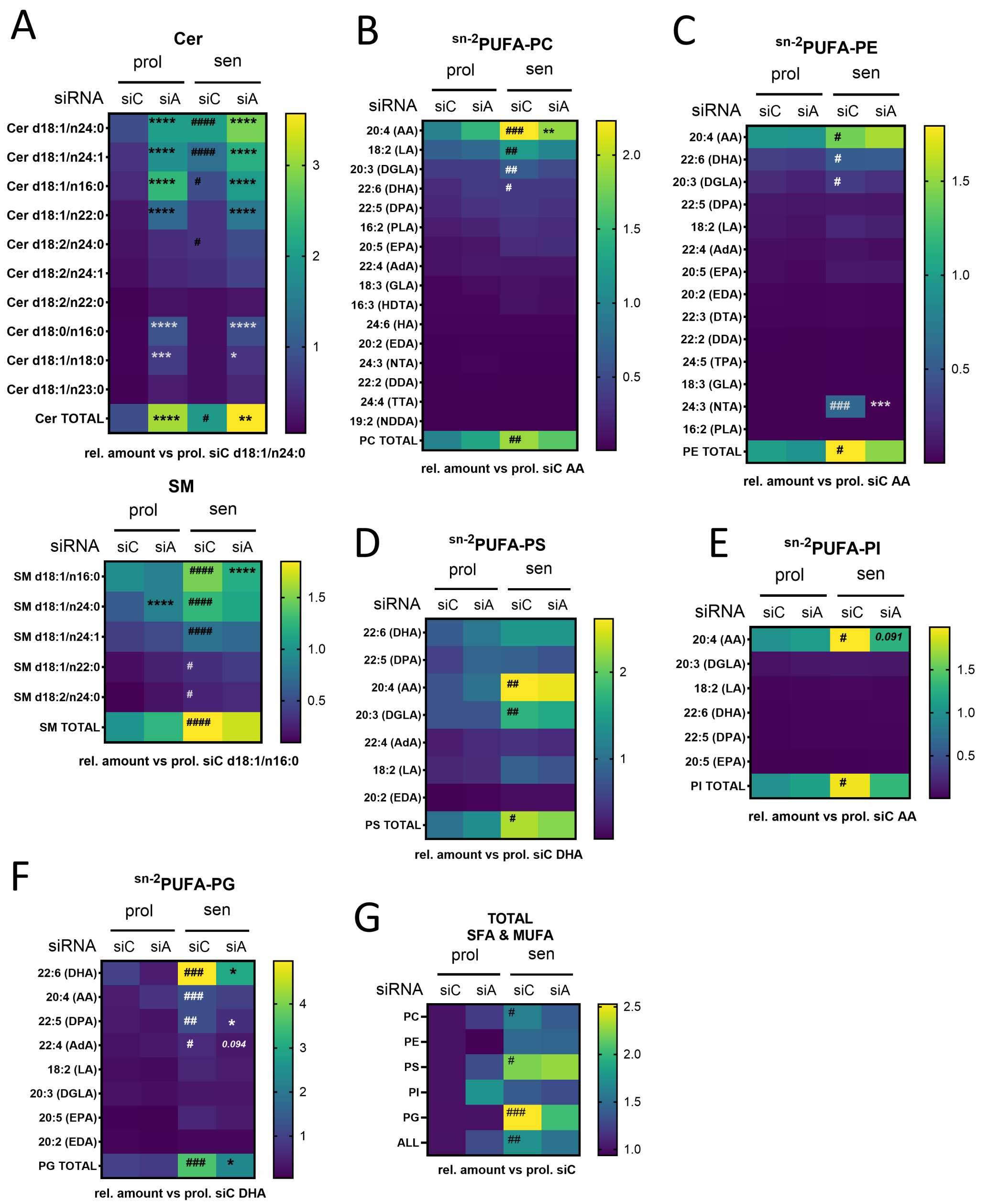

Figure S3. Heatmaps showing relative levels of the indicated lipid species compared to most abundant lipid in proliferative siCTRL cells. * indicates statistical significance between siCTRL and siACase in prol or sen cells. # indicates statistical significance between prol siCTRL and sen siCTRL. ($^{*/\#}p < 0.05$, $^{**/\#\#}p < 0.01$, $^{***/\#\#\#}p < 0.001$, $^{****/\#\#\#\#}p < 0.0001$. One-way ANOVA). prol, proliferative; sen, senescent; siC, CTRL siRNA; siA, ACase siRNA; Cer, ceramide; SM, sphingomyelin; SFA/MUFA, saturated fatty acids/monounsaturated fatty acids; PUFA, polyunsaturated fatty acid; AA, arachidonic acid; DHA, docosahexaenoic acid; DPA, docosapentanoic acid; DGLA, dihomo-gamma-linoleic acid; LA, linoleic acid; AdA, adrenic acid; EDA, eicosadienoic acid; PLA, palmitolinoleic acid; EPA, eicosapentaenoic acid; GLA, gamma-linoleic acid; HDTA, hexadecatrienoic acid; HA, herring acid; NTA, nervionic trienoic acid; DDA, docosadienoic acid; TTA, tetracosatetranoic acid; NDDA, nonadecatrienoic acid; PC, phosphatidylcholine; PE, phosphatidylethanolamine; PS, phosphatidylserine; PI, phosphatidylinositol; PG, phosphatidylglycerol.

Figure S4

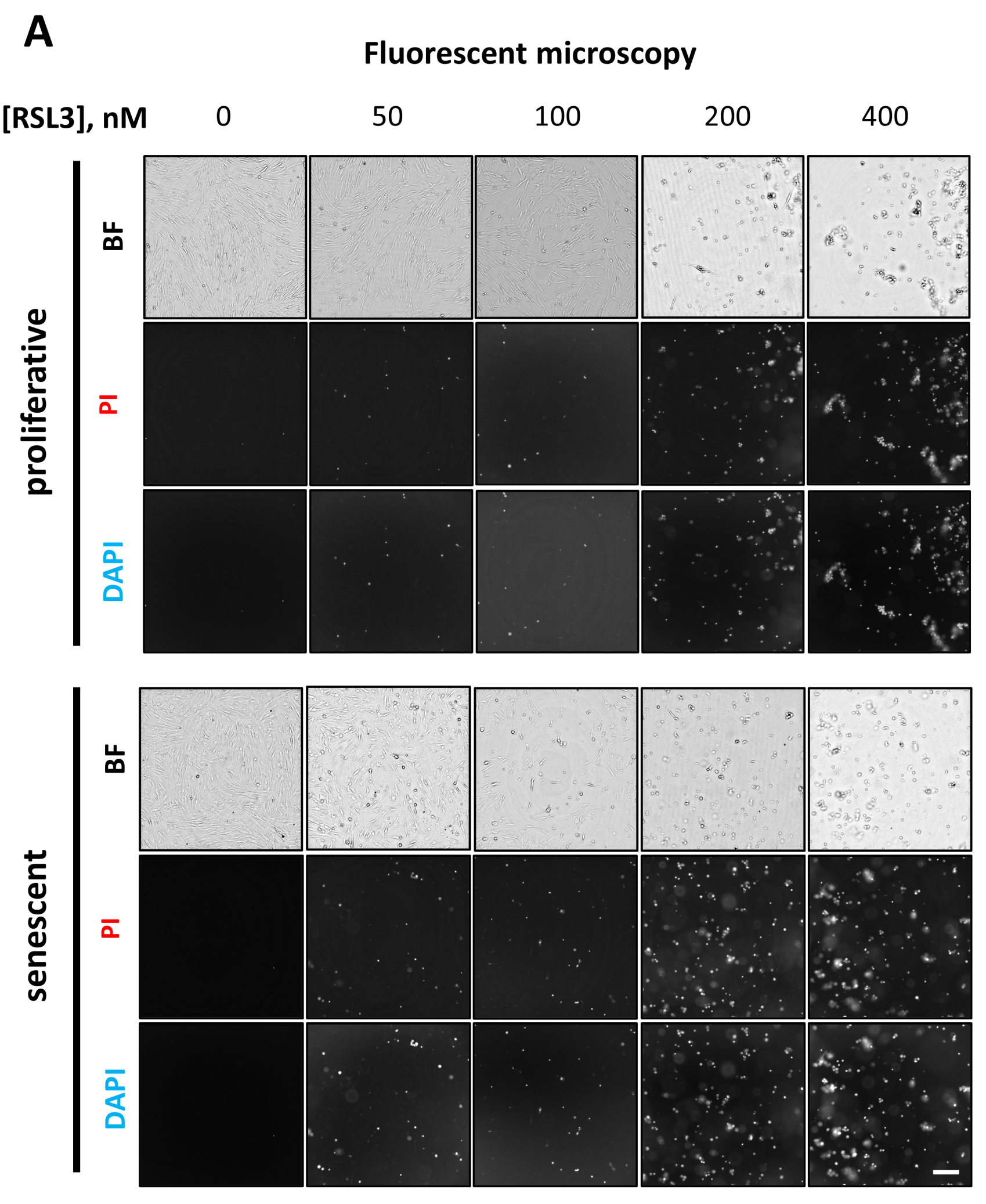
A
Fluorescent microscopy
[RSL3], nM
0
50
100
200
400
proliferative
BF
PI
DAPI
senescent
BF
PI
DAPI

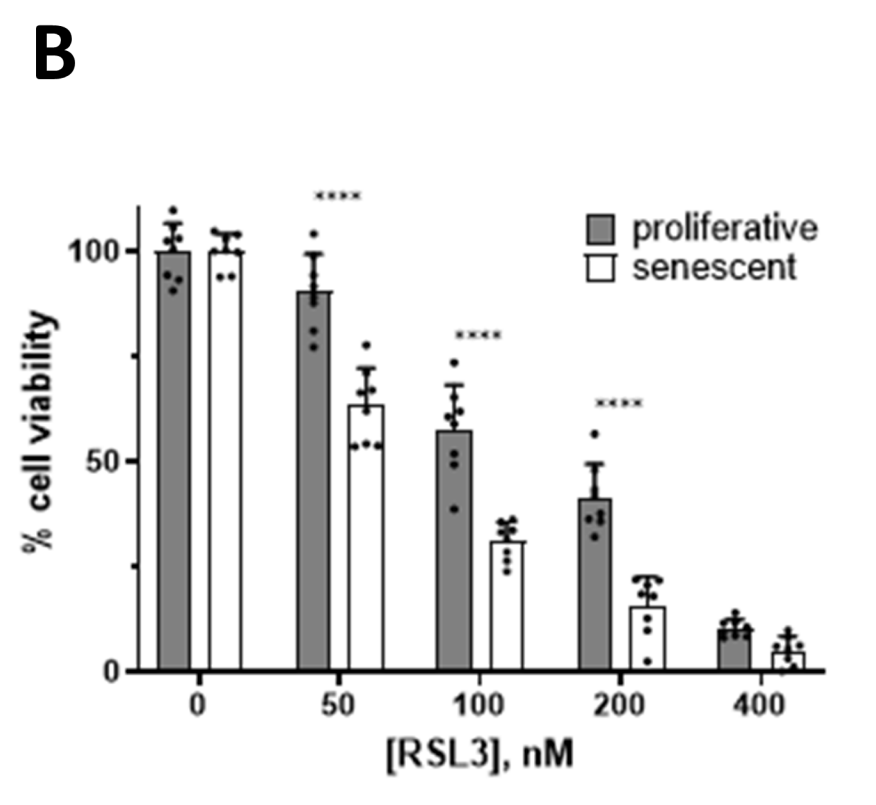
B
% cell viability
100
50
0
****
****
****
proliferative
senescent
0
50
100
200
400
[RSL3], nM

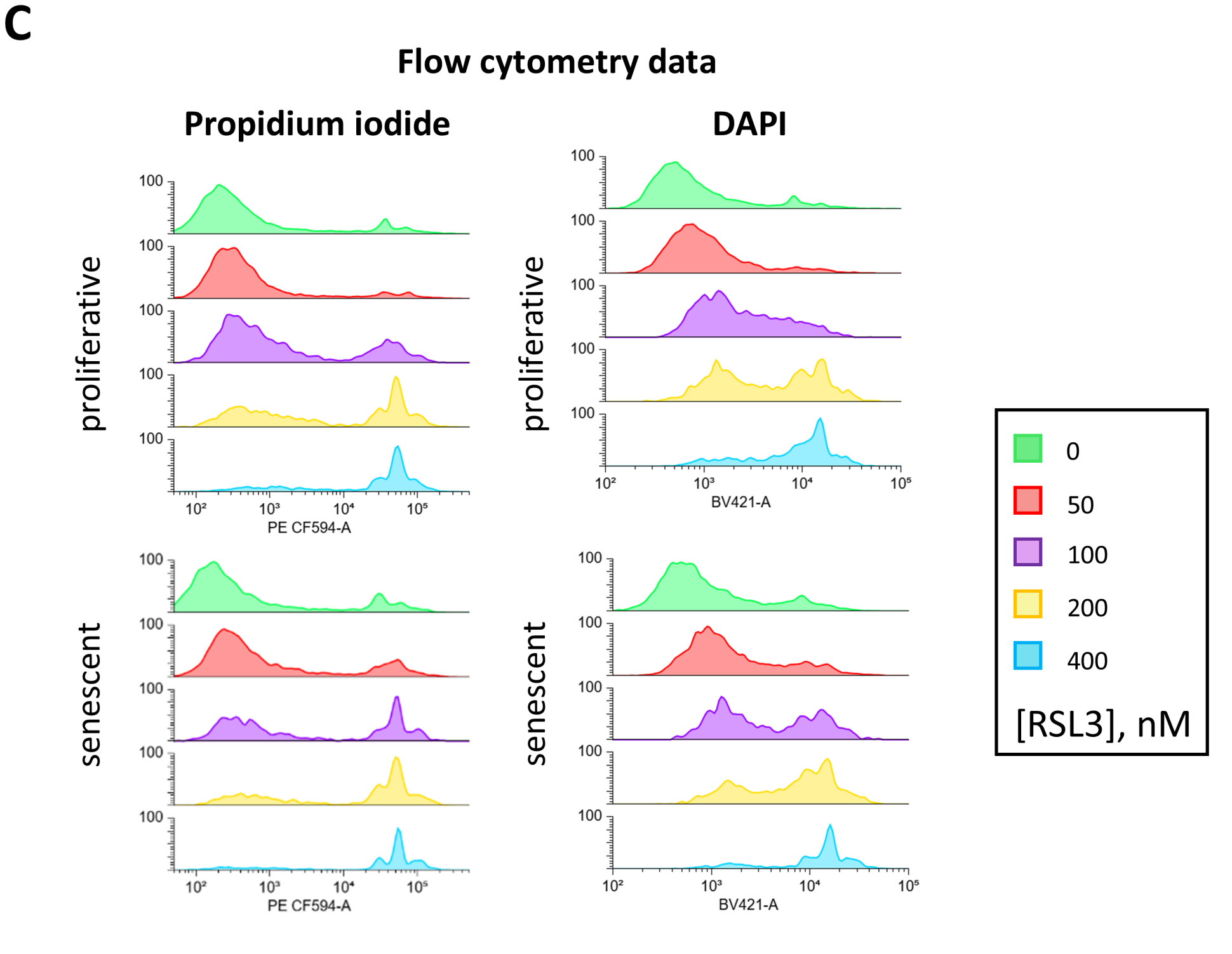
C
Flow cytometry data
Propidium iodide
DAPI
proliferative
senescent
100
PE CF594-A
BV421-A
0
50
100
200
400
[RSL3], nM

Figure S4. MTT cell viability data is consistent with DAPI and PI data. (A) Mircrographs showing brightfield (BF), propidium iodide (PI) (fluorescent emission 618 nm) and DAPI (fluorescent emission 461 nm) images of proliferative and senescent cells after treatment with indicated concentrations of RSL3 (14h). (B) percentage of cell viability measured as MTT spectrophotometric signal after treatment with indicated concentrations of RSL3 (14h). (C) Flow cytometry histograms showing PI and DAPI fluorescent emission after treatment with indicated concentrations of RSL3 (14h). ($^{****}p < 0.0001$. Two-way ANOVA). Values represent the mean ± SEM of 3 independent experiments. BF, bright field; PI, propidium iodide. Bar, 100 μm.

Supplementary Fig. S5

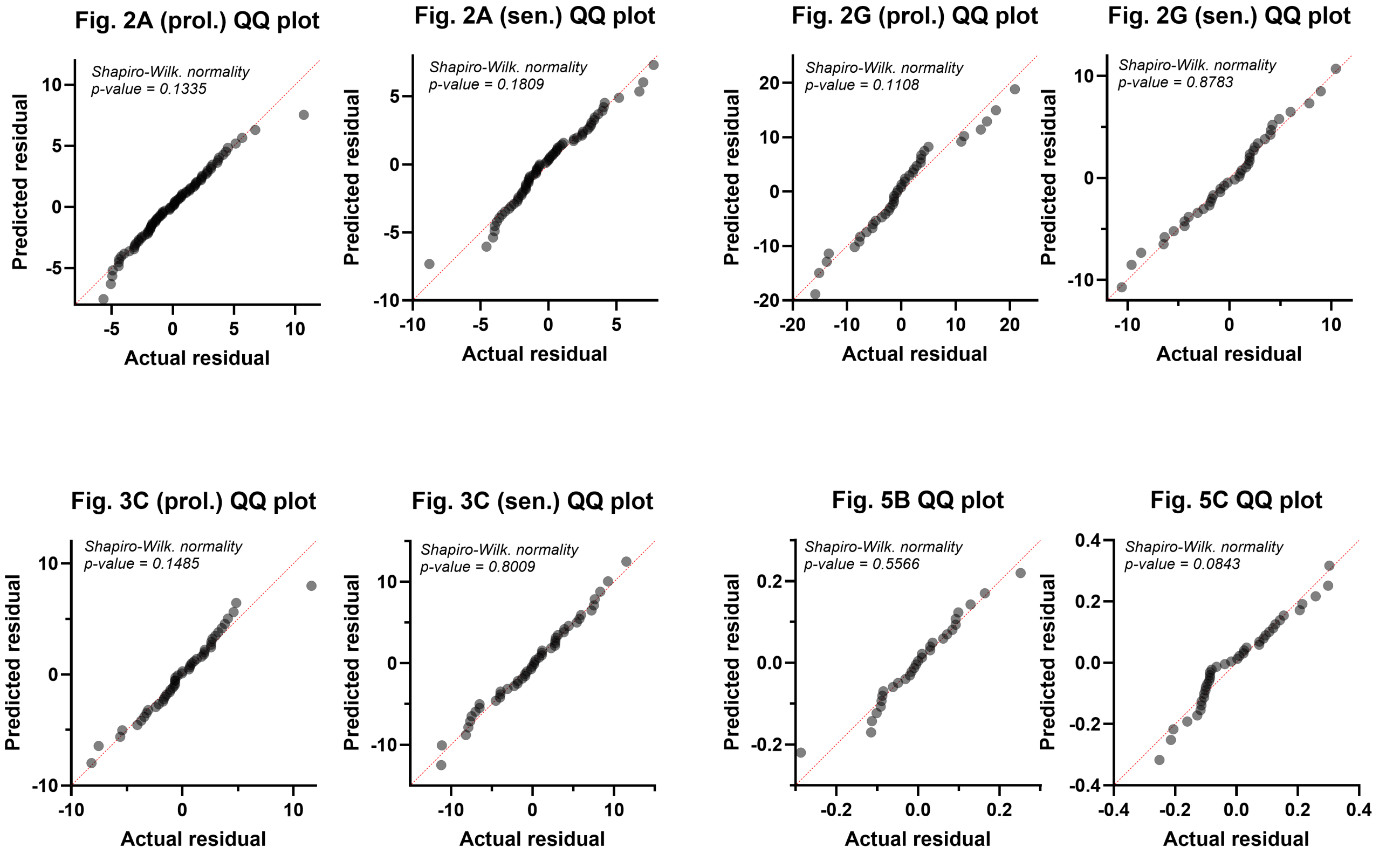

Figure S5. Normality of two-way ANOVA residuals for dose-response panels assessed by QQ plot. All plots confirm approximately normally distributed residuals (p-value ≥ 0.05).

Supplementary table S1

Figure	*Panel*	*Group/condition*	*n*	*Shapiro-Wilk. w*	*Shapiro-Wilk. p*	*Passed (α≥0.05)?*
Fig. 1	C	UT	N/A	N/A	N/A	N/A
		PDL 20	6	0.9342	0.6133	Yes
		PDL 35	6	0.9772	0.9367	Yes
		PDL 45	6	0.9473	0.7186	Yes
		Sen.	6	0.8728	0.2378	Yes
	D	Prol. DMSO	4	0.8402	0.1961	Yes
		Prol. J147	4	0.8808	0.3421	Yes
		Prol. RSL3	4	0.9830	0.9192	Yes
		Prol. RSL3+J147	4	0.9391	0.6489	Yes
		Sen. DMSO	3	0.7747	0.0554	Yes
		Sen. J147	3	0.9989	0.9370	Yes
		Sen. RSL3	3	0.9981	0.9164	Yes
		Sen. RSL3+J147	3	0.9868	0.7804	Yes
	F	Prol. DMSO	7	0.9405	0.6430	Yes
		Prol. RSL3	5	0.9069	0.3750	Yes
		Sen. DMSO	7	0.8568	0.1416	Yes
		Sen. RSL3	5	0.8889	0.3516	Yes
Fig. 2	F IL-6	Prol. siC	6	0.9566	0.7931	Yes
		Prol. siA	6	0.9550	0.7804	Yes
		Sen. siC	7	0.9368	0.6104	Yes
		Sen. siA	7	0.8896	0.2723	Yes
	F IL-8	Prol. siC	5	0.8848	0.3316	Yes
		Prol. siA	4	0.8891	0.3789	Yes
		Sen. siC	7	0.9076	0.3795	Yes
		Sen. siA	7	0.8868	0.2581	Yes
	I prol.	dmso siC	4	0.9447	0.6830	Yes
		dmso siA	3	0.8252	0.1762	Yes
		RSL3 siC	4	0.8793	0.3357	Yes
		RSL3 siA	3	0.8341	0.1989	Yes
	I sen.	dmso siC	5	0.8862	0.3385	Yes
		dmso siA	4	0.7473	0.0365	No
		RSL3 siC	4	0.7909	0.0929	Yes
		RSL3 siA	5	0.8160	0.1088	Yes
Fig. 3	B prol.	siCTRL ctrl	4	N/A	N/A	N/A
		siCTRL rsl3	3	0.9993	0.9484	Yes
		siCTRL BSO	3	N/A	N/A	N/A
		siACase ctrl	4	0.7722	0.0610	Yes
		siACase rsl3	3	0.9993	0.9480	Yes
		siACase BSO	3	N/A	N/A	N/A
	B sen.	siCTRL ctrl	5	N/A	N/A	N/A
		siCTRL rsl3	4	0.9994	0.9984	Yes
		siCTRL BSO	4	0.8808	0.3268	Yes
		siACase ctrl	4	0.8918	0.3914	Yes
		siACase rsl3	4	0.8904	0.3850	Yes
		siACase BSO	3	0.8278	0.1828	Yes
	F	prol. siCTRL	6	0.9210	0.5365	Yes
		prol. siACase	6	0.9168	0.5093	Yes
		sen. siCTRL	6	0.9254	0.5447	Yes
		sen. siACase	6	0.8318	0.1113	Yes
Fig. 4 (normality of residuals, Shapiro-wilk.)	B	N/A	N/A	0.9665	0.5817	Yes
	C	PC (AA)	N/A	0.9769	0.8320	Yes
		PE (AA)	N/A	0.9436	0.1965	Yes
		PS (DHA)	N/A	0.9562	0.3663	Yes
		PI (AA)	N/A	0.9813	0.9193	Yes
		PG (DHA)	N/A	0.9548	0.3429	Yes
Fig. 5	D	Ctl ins. dmso	4	0.9714	0.8500	Yes
		Ctl ins. Rsl3	4	0.8418	0.2007	Yes
		Sen. ins. dmso	4	0.9729	0.8594	Yes
		Sen. ins. Rsl3	4	0.9137	0.5022	Yes
	E	Ctl ins. dmso	4	0.9328	0.6110	Yes
		Ctl ins. Rsl3	4	0.9803	0.9038	Yes
		Sen. ins. dmso	4	0.8274	0.1612	Yes
		Sen. ins. Rsl3	4	0.8406	0.1972	Yes
	F	Ctl. dmso	4	0.9206	0.5402	Yes
		Ctl. Rsl3	5	0.9237	0.5538	Yes
		IL6/8 dmso	5	0.9751	0.9066	Yes
		IL6/8 Rsl3	5	0.9530	0.7583	Yes
	G	Ctl. dmso	4	0.9167	0.5186	Yes
		Ctl. Rsl3	4	0.9815	0.9109	Yes
		IL6/8 dmso	4	0.8700	0.2975	Yes
		IL6/8 Rsl3	4	0.9406	0.6580	Yes
Fig. S1	A	Ctl dmso	4	0.9075	0.4691	Yes
		ARN14 dmso	4	0.8318	0.1725	Yes
		Ctl rsl3	4	0.8241	0.1528	Yes
		ARN14 rsl3	4	0.9372	0.6376	Yes
	B	Ctl dmso	4	0.9380	0.6420	Yes
		ARN14 dmso	4	0.8652	0.2793	Yes
		Ctl rsl3	4	0.8380	0.1896	Yes
		ARN14 rsl3	4	0.7091	0.0147	No
	C	Ctl dmso	4	0.8635	0.2731	Yes
		ARN14 dmso	3	0.9009	0.3883	Yes
		Ctl rsl3	4	0.9209	0.5419	Yes
		ARN14 rsl3	3	0.9957	0.8743	Yes

Table S1. Shapiro-Wilk normality test results for all quantitative datasets. For dose-response panels, normality of two-way ANOVA residuals was assessed by QQ plot (Supplementary Figure S3). p-value ≥ 0.05 indicate that normality cannot be rejected. N/A, not applicable or insufficient sample size for testing.
