# Supplementary material for: Acid ceramidase modulates the lipid profile and exacerbates sensitivity to ferroptosis in WI-38 replicative senescent cells
Source: Cell Death Dis. 2026 Jul 10;17(1):860. doi: 10.1038/s41419-026-09108-y (PMC13638989; doi:10.1038/s41419-026-09108-y)
Supplement: Supplementary file 2 [file 41419_2026_9108_MOESM3_ESM.pdf]

Fig. 2

B

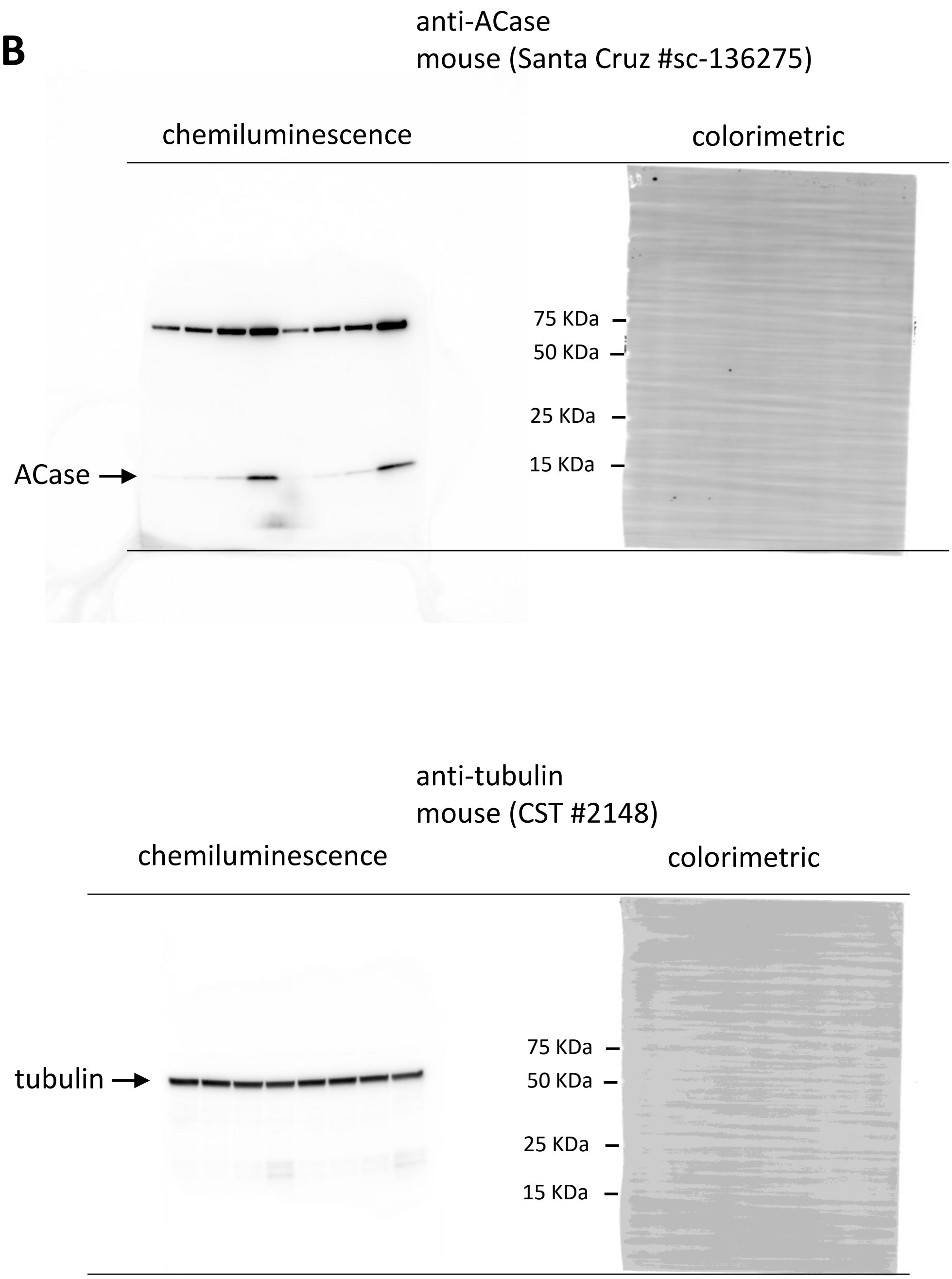

anti-ACase
mouse (Santa Cruz #sc-136275)
chemiluminescence
colorimetric
75 KDa
50 KDa
25 KDa
15 KDa
ACase
anti-tubulin
mouse (CST #2148)
chemiluminescence
colorimetric
75 KDa
50 KDa
25 KDa
15 KDa
tubulin

Fig. 2

C

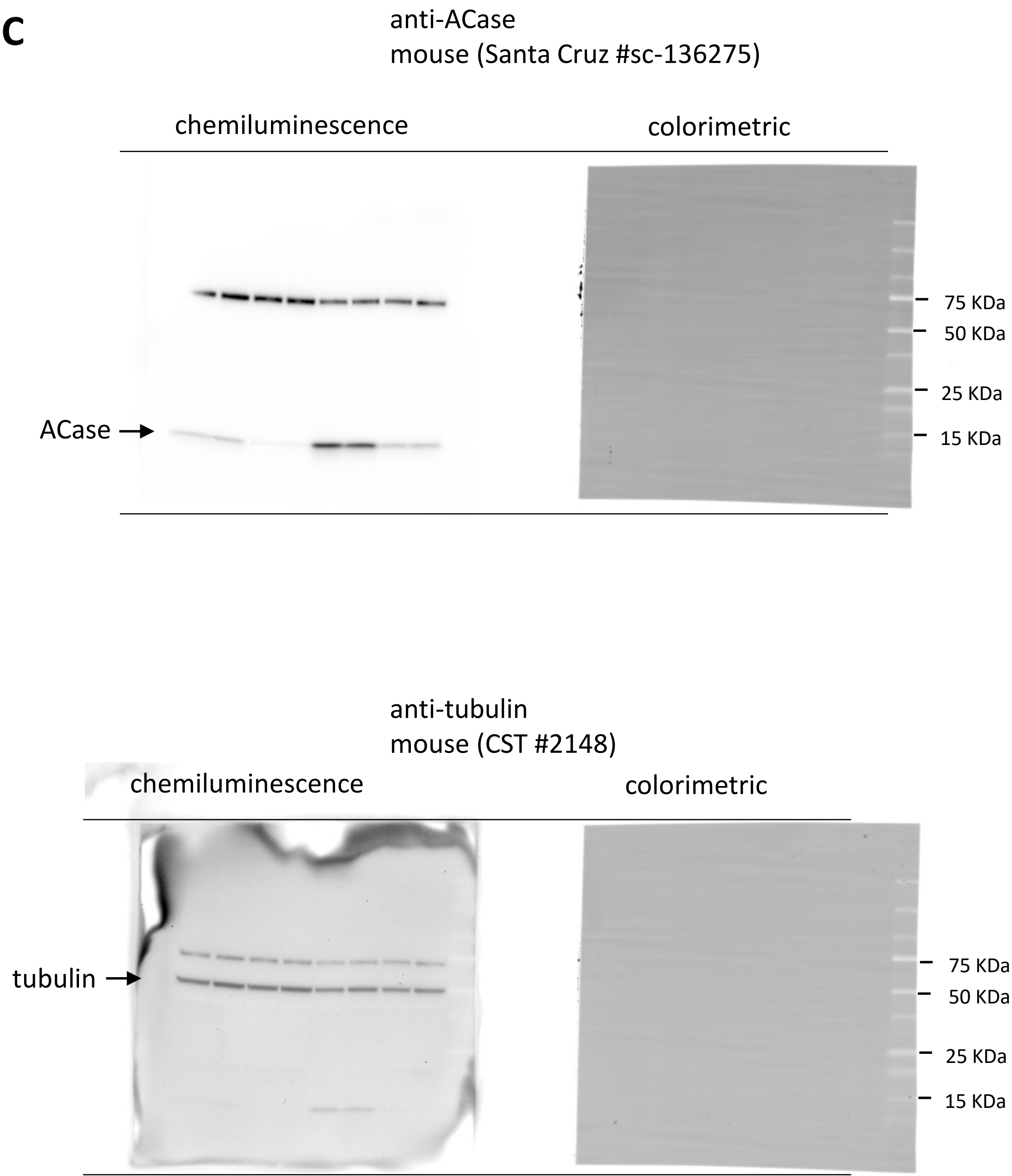
anti-ACase
mouse (Santa Cruz #sc-136275)
chemiluminescence
colorimetric
ACase
75 KDa
50 KDa
25 KDa
15 KDa
anti-tubulin
mouse (CST #2148)
chemiluminescence
colorimetric
tubulin
75 KDa
50 KDa
25 KDa
15 KDa

Fig. 2

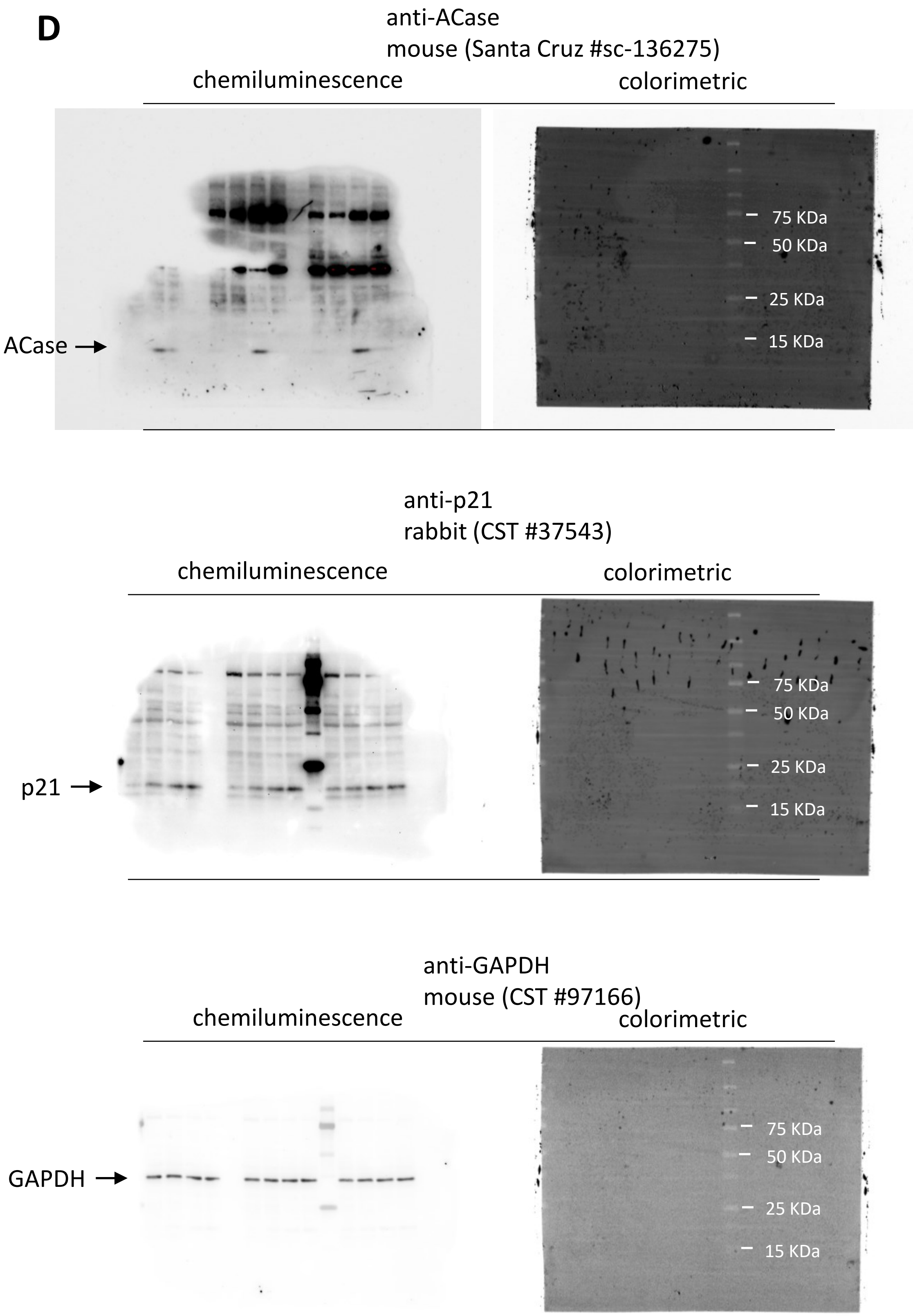
D
anti-ACase
mouse (Santa Cruz #sc-136275)
chemiluminescence
colorimetric
ACase
75 KDa
50 KDa
25 KDa
15 KDa
anti-p21
rabbit (CST #37543)
chemiluminescence
colorimetric
p21
75 KDa
50 KDa
25 KDa
15 KDa
anti-GAPDH
mouse (CST #97166)
chemiluminescence
colorimetric
GAPDH
75 KDa
50 KDa
25 KDa
15 KDa

Fig. 3

D

anti-ACase
mouse (Santa Cruz #sc-136275)

chemiluminescence | colorimetric

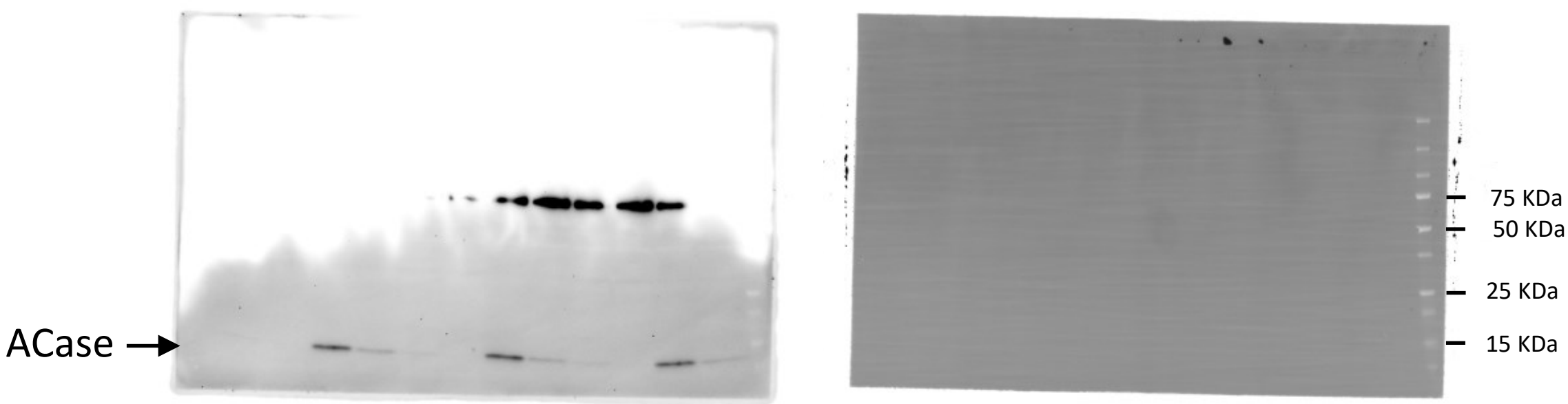

anti-GPX4
mouse (Santa Cruz #166570)

chemiluminescence | colorimetric

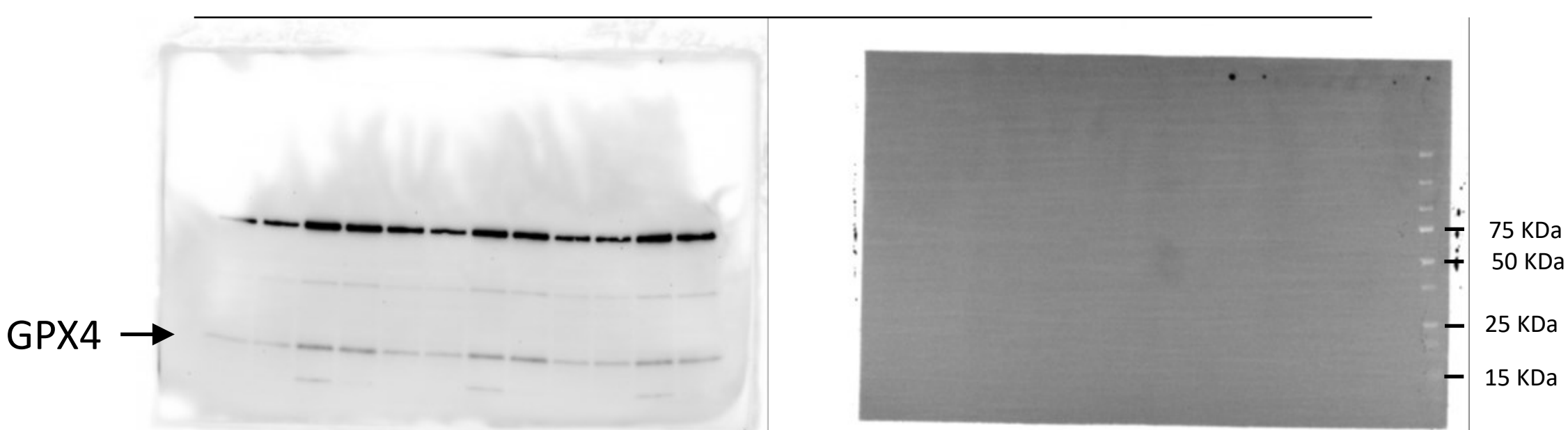

anti-tubulin
mouse (CST #2148)

chemiluminescence | colorimetric

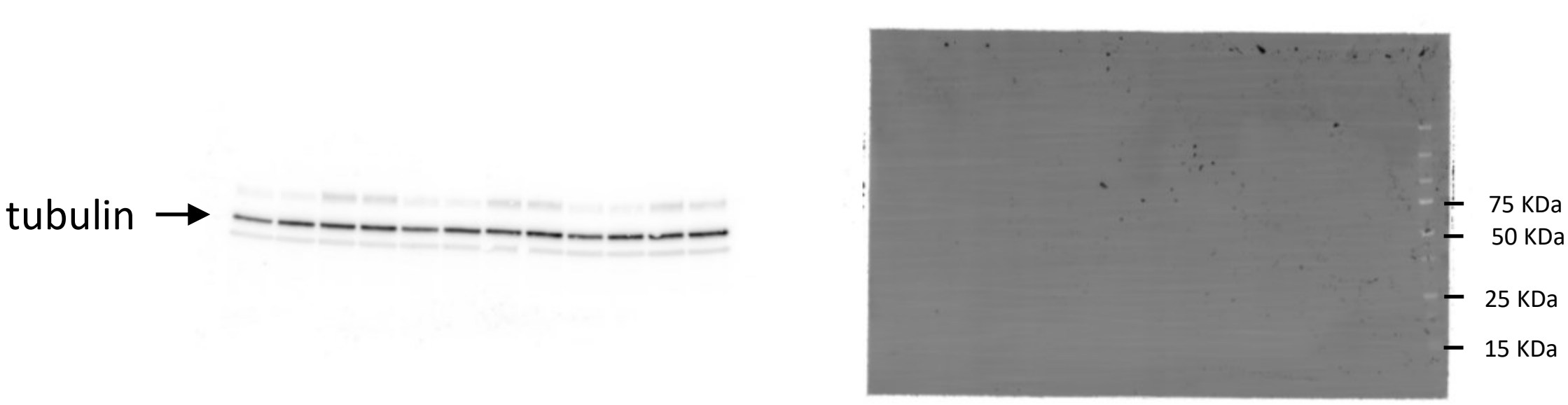

Fig. 3

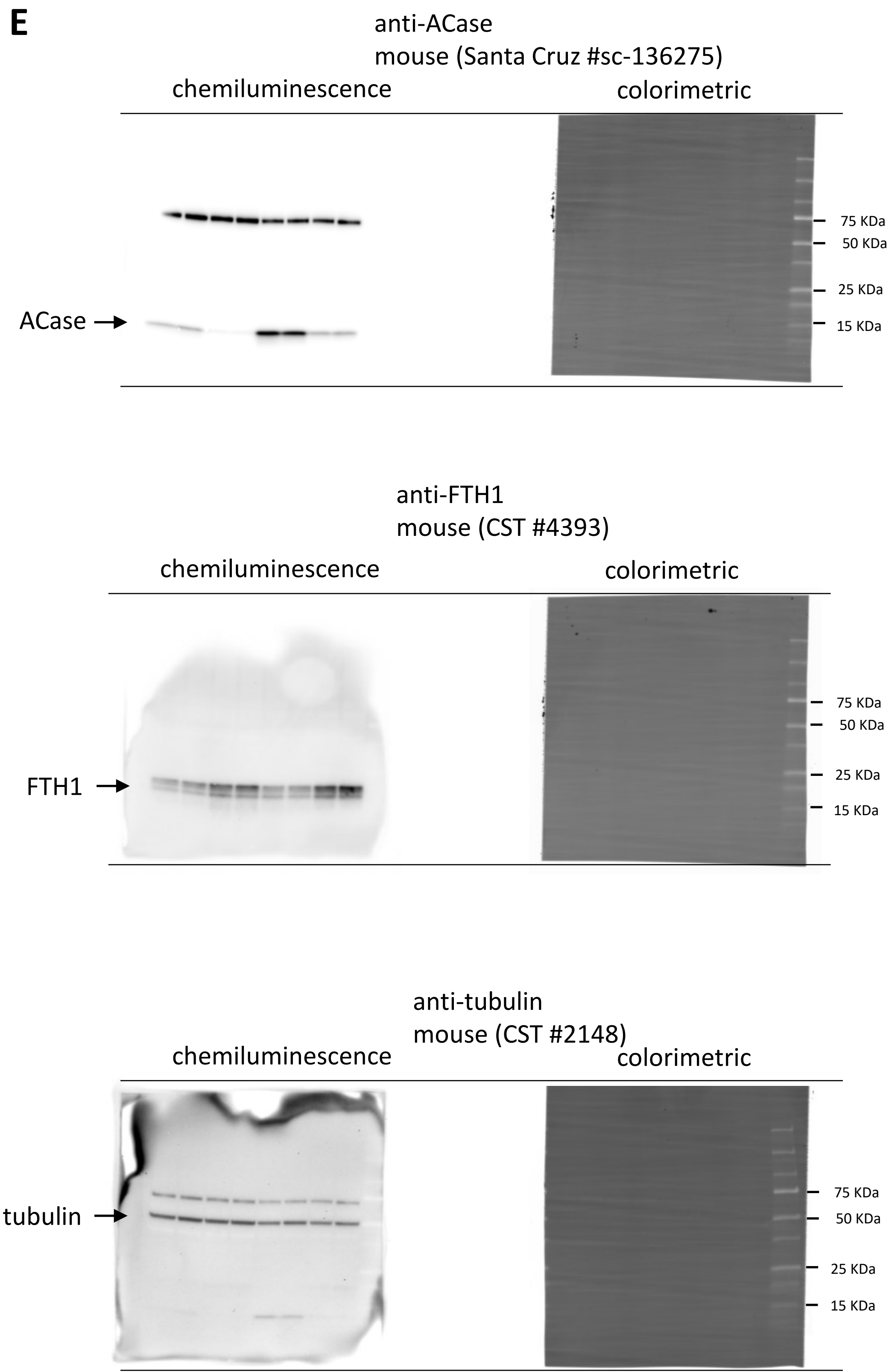
E
anti-ACase
mouse (Santa Cruz #sc-136275)
chemiluminescence
colorimetric
ACase
75 KDa
50 KDa
25 KDa
15 KDa
anti-FTH1
mouse (CST #4393)
chemiluminescence
colorimetric
FTH1
75 KDa
50 KDa
25 KDa
15 KDa
anti-tubulin
mouse (CST #2148)
chemiluminescence
colorimetric
tubulin
75 KDa
50 KDa
25 KDa
15 KDa

Fig. 4

D

anti-ACase
mouse (Santa Cruz #sc-136275)

chemiluminescence | colorimetric

ACase →

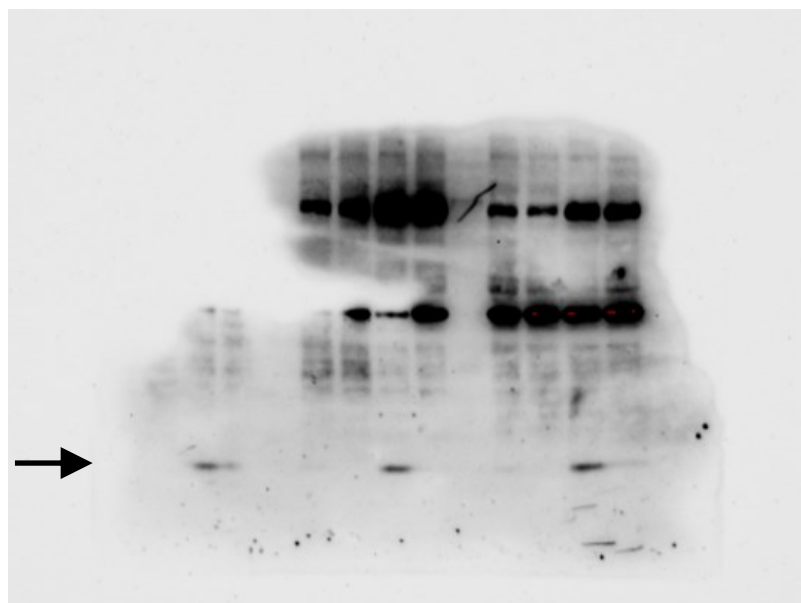

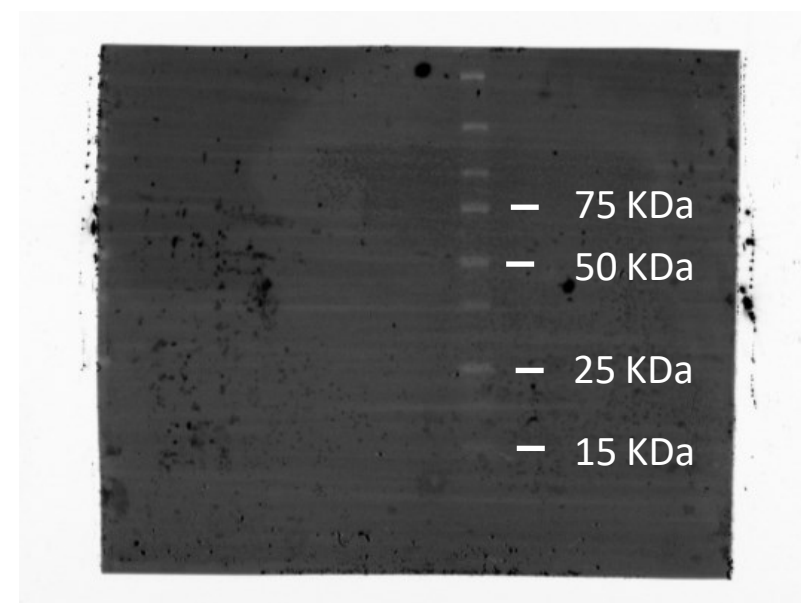

anti-ACSL4
mouse (Santa Cruz #sc-271800)

chemiluminescence | colorimetric

ACSL4 →

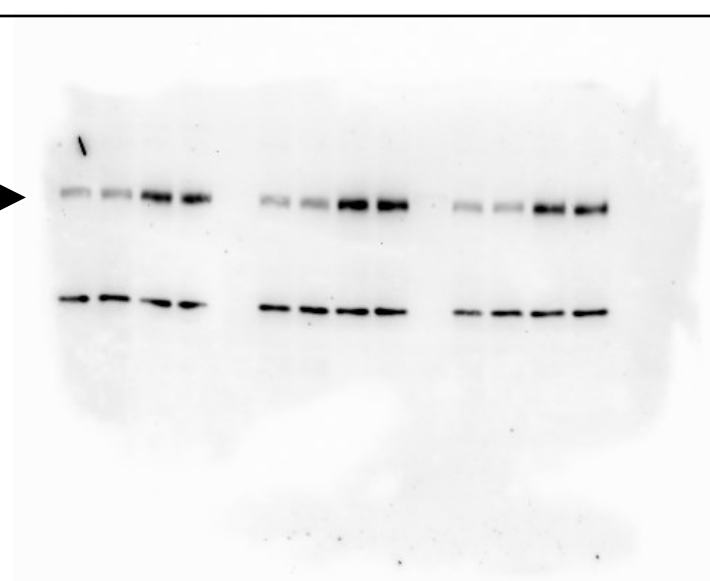

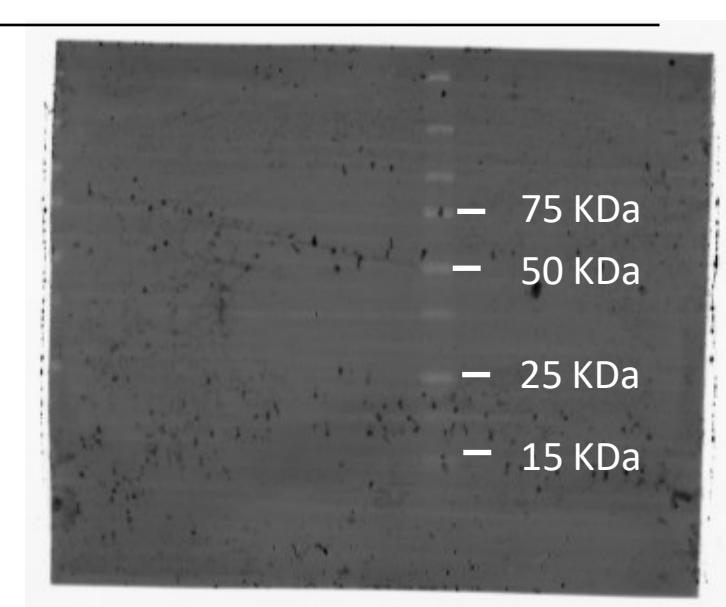

anti-GAPDH
mouse (CST #97166)

chemiluminescence | colorimetric

GAPDH →

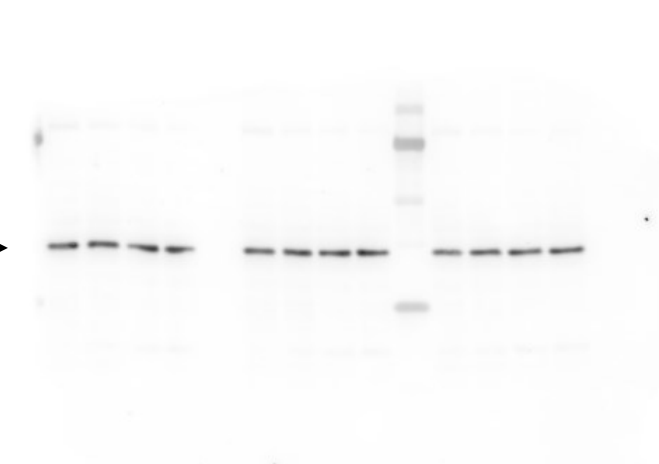

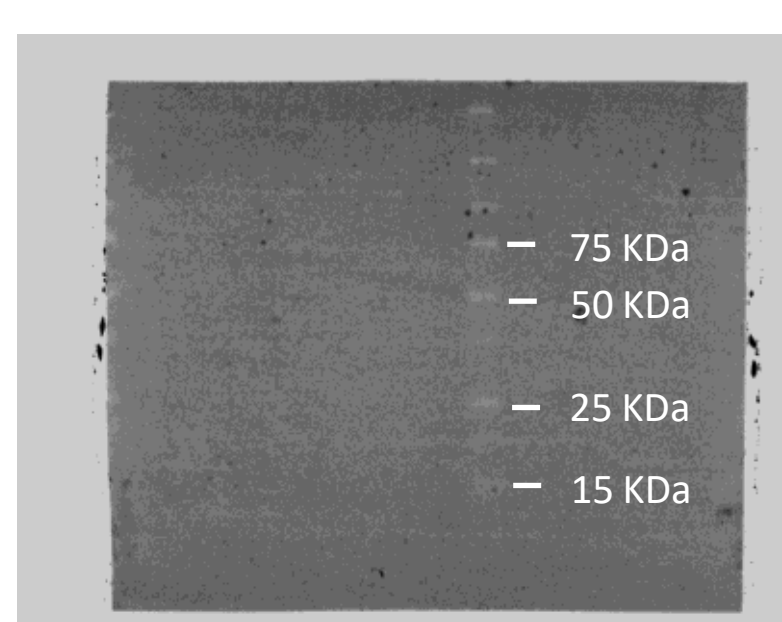

Fig. 5

B

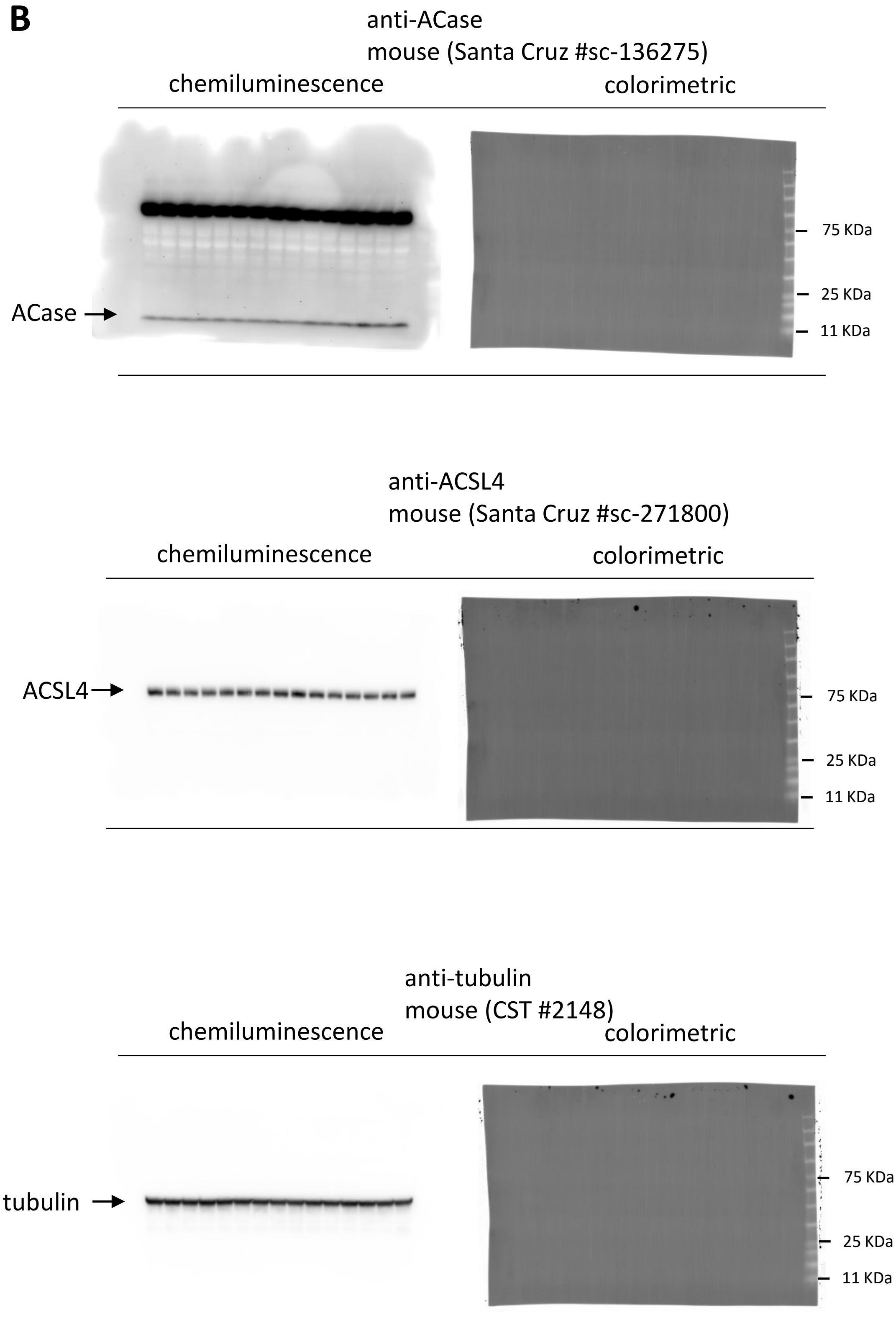
anti-ACase
mouse (Santa Cruz #sc-136275)
chemiluminescence
colorimetric
ACase
75 KDa
25 KDa
11 KDa
anti-ACSL4
mouse (Santa Cruz #sc-271800)
chemiluminescence
colorimetric
ACSL4
75 KDa
25 KDa
11 KDa
anti-tubulin
mouse (CST #2148)
chemiluminescence
colorimetric
tubulin
75 KDa
25 KDa
11 KDa

Fig. 5

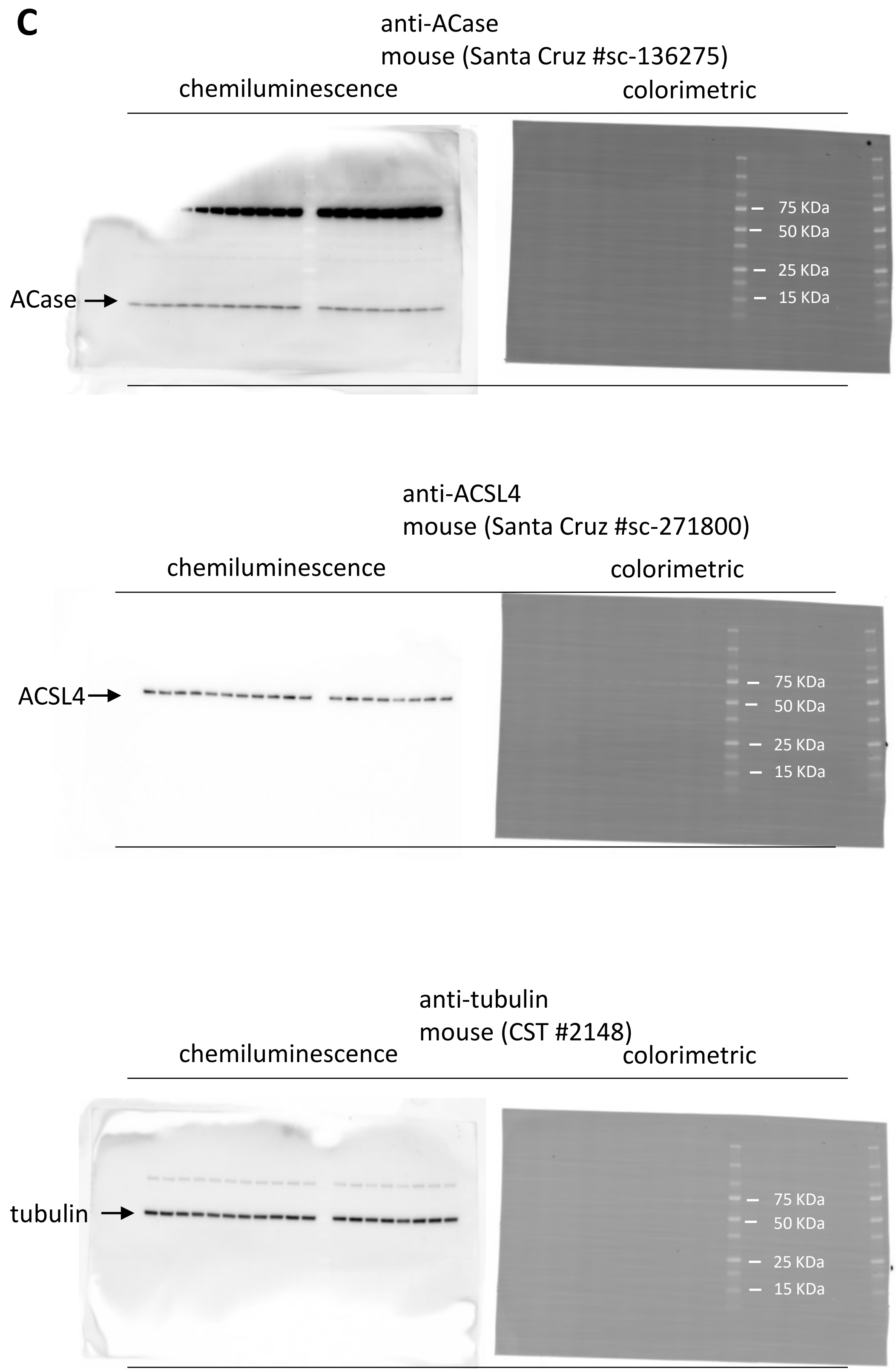
C
anti-ACase
mouse (Santa Cruz #sc-136275)
chemiluminescence
colorimetric
ACase
75 KDa
50 KDa
25 KDa
15 KDa
anti-ACSL4
mouse (Santa Cruz #sc-271800)
chemiluminescence
colorimetric
ACSL4
75 KDa
50 KDa
25 KDa
15 KDa
anti-tubulin
mouse (CST #2148)
chemiluminescence
colorimetric
tubulin
75 KDa
50 KDa
25 KDa
15 KDa

Supplementary Fig. S1

anti-ACase
mouse (Santa Cruz #sc-136275)

chemiluminescence | colorimetric

ACase →

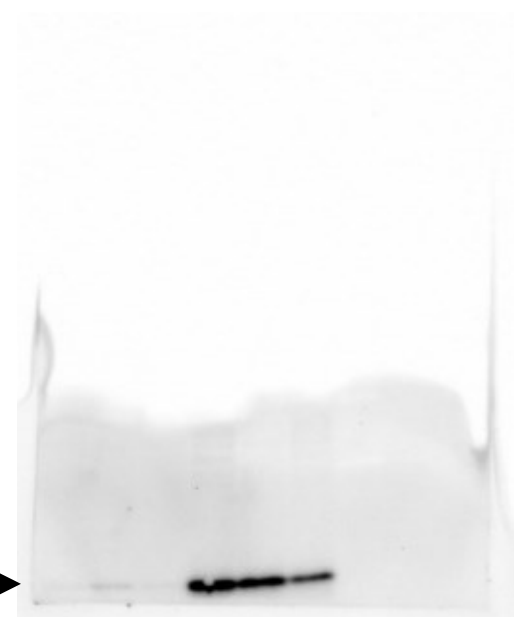

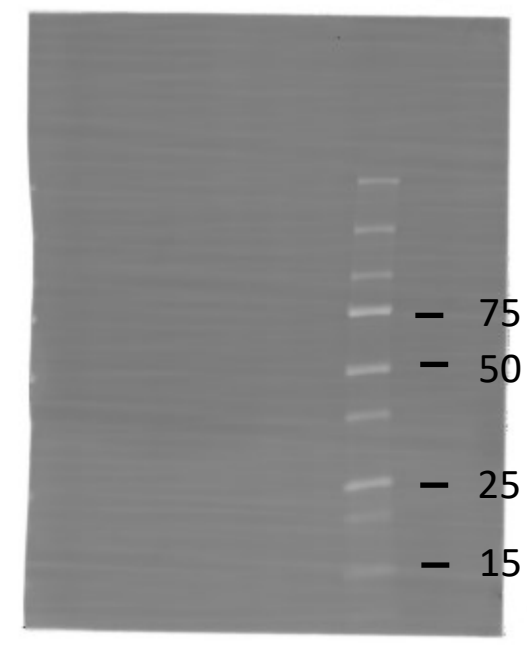

75 KDa
50 KDa
25 KDa
15 KDa

anti-ACSL4
mouse (Santa Cruz #sc-271800)

chemiluminescence | colorimetric

ACSL4 →

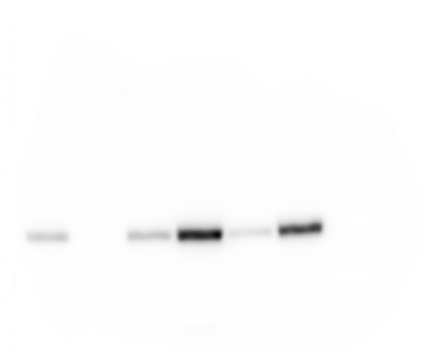

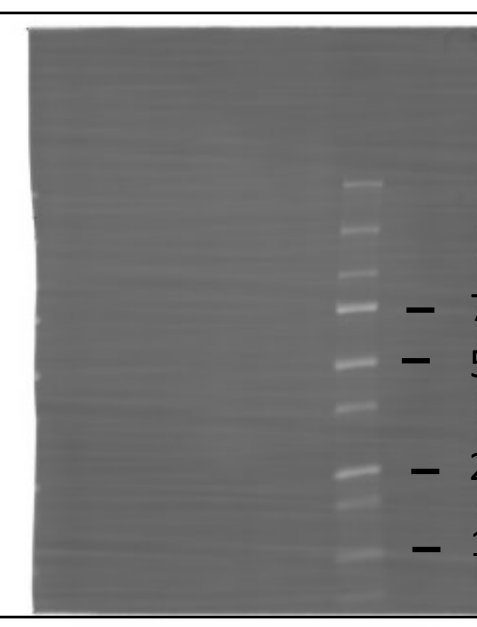

75 KDa
50 KDa
25 KDa
15 KDa

4-20% Criterion TGX Stain-Free (Bio-Rad #5678094)

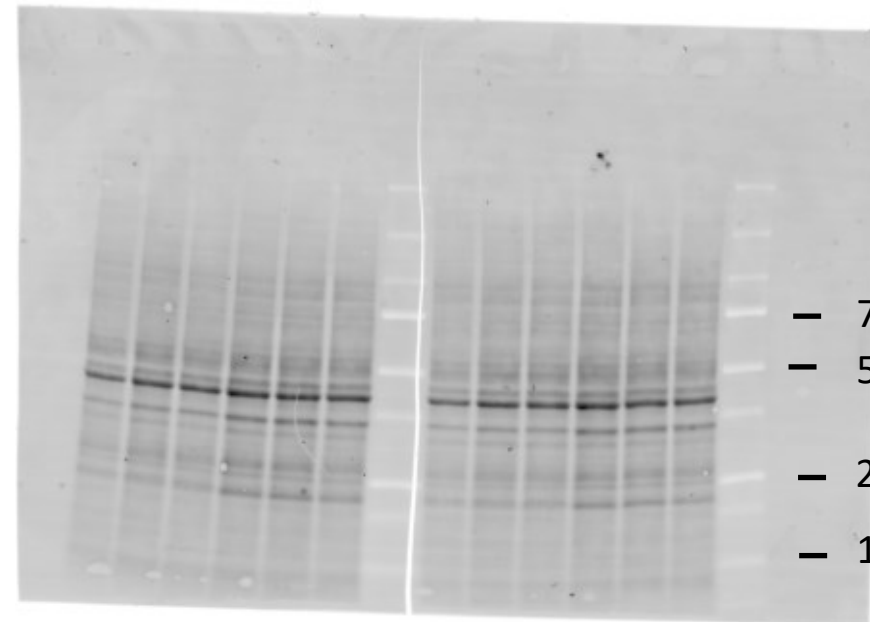

75 KDa
50 KDa
25 KDa
15 KDa
