# Supplementary material for: Acid ceramidase modulates the lipid profile and exacerbates sensitivity to ferroptosis in WI-38 replicative senescent cells
Source: Cell Death Dis. 2026 Jul 10;17(1):860. doi: 10.1038/s41419-026-09108-y (PMC13638989; doi:10.1038/s41419-026-09108-y)
Supplement: Supplementary file 3 [file 41419_2026_9108_MOESM2_ESM.docx]

| ***Figure*** | ***Panel*** | ***Group/condition*** | ***n*** | ***Shapiro-Wilk. w*** | ***Shapiro-Wilk. p*** | ***Passed (α≥0.05)?*** |
| --- | --- | --- | --- | --- | --- | --- |
| ***Fig. 1*** | C | UT | N/A | N/A | N/A | N/A |
|  |  | PDL 20 | 6 | 0.9342 | 0.6133 | Yes |
|  |  | PDL 35 | 6 | 0.9772 | 0.9367 | Yes |
|  |  | PDL 45 | 6 | 0.9473 | 0.7186 | Yes |
|  |  | Sen. | 6 | 0.8728 | 0.2378 | Yes |
|  | D | Prol. DMSO | 4 | 0.8402 | 0.1961 | Yes |
|  |  | Prol. J147 | 4 | 0.8808 | 0.3421 | Yes |
|  |  | Prol. RSL3 | 4 | 0.9830 | 0.9192 | Yes |
|  |  | Prol. RSL3+J147 | 4 | 0.9391 | 0.6489 | Yes |
|  |  | Sen. DMSO | 3 | 0.7747 | 0.0554 | Yes |
|  |  | Sen. J147 | 3 | 0.9989 | 0.9370 | Yes |
|  |  | Sen. RSL3 | 3 | 0.9981 | 0.9164 | Yes |
|  |  | Sen. RSL3+J147 | 3 | 0.9868 | 0.7804 | Yes |
|  | F | Prol. DMSO | 7 | 0.9405 | 0.6430 | Yes |
|  |  | Prol. RSL3 | 5 | 0.9069 | 0.3750 | Yes |
|  |  | Sen. DMSO | 7 | 0.8568 | 0.1416 | Yes |
|  |  | Sen. RSL3 | 5 | 0.8889 | 0.3516 | Yes |
| ***Fig. 2*** | F IL-6 | Prol. siC | 6 | 0.9566 | 0.7931 | Yes |
|  |  | Prol. siA | 6 | 0.9550 | 0.7804 | Yes |
|  |  | Sen. siC | 7 | 0.9368 | 0.6104 | Yes |
|  |  | Sen. siA | 7 | 0.8896 | 0.2723 | Yes |
|  | F IL-8 | Prol. siC | 5 | 0.8848 | 0.3316 | Yes |
|  |  | Prol. siA | 4 | 0.8891 | 0.3789 | Yes |
|  |  | Sen. siC | 7 | 0.9076 | 0.3795 | Yes |
|  |  | Sen. siA | 7 | 0.8868 | 0.2581 | Yes |
|  | I prol. | dmso siC | 4 | 0.9447 | 0.6830 | Yes |
|  |  | dmso siA | 3 | 0.8252 | 0.1762 | Yes |
|  |  | RSL3 siC | 4 | 0.8793 | 0.3357 | Yes |
|  |  | RSL3 siA | 3 | 0.8341 | 0.1989 | Yes |
|  | I sen. | dmso siC | 5 | 0.8862 | 0.3385 | Yes |
|  |  | dmso siA | 4 | 0.7473 | 0.0365 | No |
|  |  | RSL3 siC | 4 | 0.7909 | 0.0929 | Yes |
|  |  | RSL3 siA | 5 | 0.8160 | 0.1088 | Yes |
| ***Fig. 3*** | B prol. | siCTRL ctrl | 4 | N/A | N/A | N/A |
|  |  | siCTRL rsl3 | 3 | 0.9993 | 0.9484 | Yes |
|  |  | siCTRL BSO | 3 | N/A | N/A | N/A |
|  |  | siACase ctrl | 4 | 0.7722 | 0.0610 | Yes |
|  |  | siACase rsl3 | 3 | 0.9993 | 0.9480 | Yes |
|  |  | siACase BSO | 3 | N/A | N/A | N/A |
|  | B sen. | siCTRL ctrl | 5 | N/A | N/A | N/A |
|  |  | siCTRL rsl3 | 4 | 0.9994 | 0.9984 | Yes |
|  |  | siCTRL BSO | 4 | 0.8808 | 0.3268 | Yes |
|  |  | siACase ctrl | 4 | 0.8918 | 0.3914 | Yes |
|  |  | siACase rsl3 | 4 | 0.8904 | 0.3850 | Yes |
|  |  | siACase BSO | 3 | 0.8278 | 0.1828 | Yes |
|  | F | prol. siCTRL | 6 | 0.9210 | 0.5365 | Yes |
|  |  | prol. siACase | 6 | 0.9168 | 0.5093 | Yes |
|  |  | sen. siCTRL | 6 | 0.9254 | 0.5447 | Yes |
|  |  | sen. siACase | 6 | 0.8318 | 0.1113 | Yes |
| ***Fig. 4 (normality of residuals, Shapiro-wilk.)*** | B | N/A | N/A | 0.9665 | 0.5817 | Yes |
|  | C | PC (AA) | N/A | 0.9769 | 0.8320 | Yes |
|  |  | PE (AA) | N/A | 0.9436 | 0.1965 | Yes |
|  |  | PS (DHA) | N/A | 0.9562 | 0.3663 | Yes |
|  |  | PI (AA) | N/A | 0.9813 | 0.9193 | Yes |
|  |  | PG (DHA) | N/A | 0.9548 | 0.3429 | Yes |
| ***Fig. 5*** | D | Ctl ins. dmso | 4 | 0.9714 | 0.8500 | Yes |
|  |  | Ctl ins. Rsl3 | 4 | 0.8418 | 0.2007 | Yes |
|  |  | Sen. ins. dmso | 4 | 0.9729 | 0.8594 | Yes |
|  |  | Sen. ins. Rsl3 | 4 | 0.9137 | 0.5022 | Yes |
|  | E | Ctl ins. dmso | 4 | 0.9328 | 0.6110 | Yes |
|  |  | Ctl ins. Rsl3 | 4 | 0.9803 | 0.9038 | Yes |
|  |  | Sen. ins. dmso | 4 | 0.8274 | 0.1612 | Yes |
|  |  | Sen. ins. Rsl3 | 4 | 0.8406 | 0.1972 | Yes |
|  | F | Ctl. dmso | 4 | 0.9206 | 0.5402 | Yes |
|  |  | Ctl. Rsl3 | 5 | 0.9237 | 0.5538 | Yes |
|  |  | IL6/8 dmso | 5 | 0.9751 | 0.9066 | Yes |
|  |  | IL6/8 Rsl3 | 5 | 0.9530 | 0.7583 | Yes |
|  | G | Ctl. dmso | 4 | 0.9167 | 0.5186 | Yes |
|  |  | Ctl. Rsl3 | 4 | 0.9815 | 0.9109 | Yes |
|  |  | IL6/8 dmso | 4 | 0.8700 | 0.2975 | Yes |
|  |  | IL6/8 Rsl3 | 4 | 0.9406 | 0.6580 | Yes |
| ***Fig. S1*** | A | Ctl dmso | 4 | 0.9075 | 0.4691 | Yes |
|  |  | ARN14 dmso | 4 | 0.8318 | 0.1725 | Yes |
|  |  | Ctl rsl3 | 4 | 0.8241 | 0.1528 | Yes |
|  |  | ARN14 rsl3 | 4 | 0.9372 | 0.6376 | Yes |
|  | B | Ctl dmso | 4 | 0.9380 | 0.6420 | Yes |
|  |  | ARN14 dmso | 4 | 0.8652 | 0.2793 | Yes |
|  |  | Ctl rsl3 | 4 | 0.8380 | 0.1896 | Yes |
|  |  | ARN14 rsl3 | 4 | 0.7091 | 0.0147 | No |
|  | C | Ctl dmso | 4 | 0.8635 | 0.2731 | Yes |
|  |  | ARN14 dmso | 3 | 0.9009 | 0.3883 | Yes |
|  |  | Ctl rsl3 | 4 | 0.9209 | 0.5419 | Yes |
|  |  | ARN14 rsl3 | 3 | 0.9957 | 0.8743 | Yes |
